# A mean shift algorithm for drift correction in localization microscopy

**DOI:** 10.1016/j.bpr.2021.100008

**Published:** 2021-07-24

**Authors:** Frank J. Fazekas, Thomas R. Shaw, Sumin Kim, Ryan A. Bogucki, Sarah L. Veatch

**Affiliations:** 1 Program in Biophysics, University of Michigan, Ann Arbor, Michigan; 2 Program in Applied Physics, University of Michigan, Ann Arbor, Michigan; 3 Cellular and Molecular Biology Graduate Program, University of Michigan, Ann Arbor, Michigan

## Abstract

Single-molecule localization microscopy techniques transcend the diffraction limit of visible light by localizing isolated emitters sampled stochastically. This time-lapse imaging necessitates long acquisition times, over which sample drift can become large relative to the localization precision. Here, we present an efficient and robust method for estimating drift, using a simple peak-finding algorithm based on mean shifts that is effective for single-molecule localization microscopy in two or three dimensions.

## INTRODUCTION

Stochastic super-resolution microscopy techniques, such as STORM (1,2) and PALM (3,4), exploit photo-switching of fluorescent probes to enable imaging of densely labeled samples with resolutions an order of magnitude smaller than the diffraction limit of visible light. Sparsely distributed point spread functions (PSFs) of single emitters are identified in individual image frames, and their centroids are determined according to an appropriate fitting algorithm. The axial position of molecules can be encoded in their PSFs through engineering measures utilizing astigmatism (5,6), multifocal plane imaging (7), or a double helix PSF (8). The final reconstruction is typically a two-dimensional (2D) or three-dimensional (3D) histogram of these single-molecule positions.

Drift due to thermal expansion or mechanical instabilities can degrade image quality over the course of image acquisition, which typically occurs on the time-scale of minutes. Drift compensation requires either active stabilization of the microscope (9-13) or a posteriori computation of the drift curves, either using fiducial markers (14-18) or the acquired single-molecule localizations (19-26). In this report, we present a mathematically simple approach to drift correction using a mean shift (MS) algorithm (27-29) for static single-molecule localization microscopy (SMLM) data sets without fiducial markers, with some advantages over past approaches that use nonlinear least-squares (NLLS) fitting of image-based cross-correlations (19-21).

## RESULTS

**FIGURE 1:**
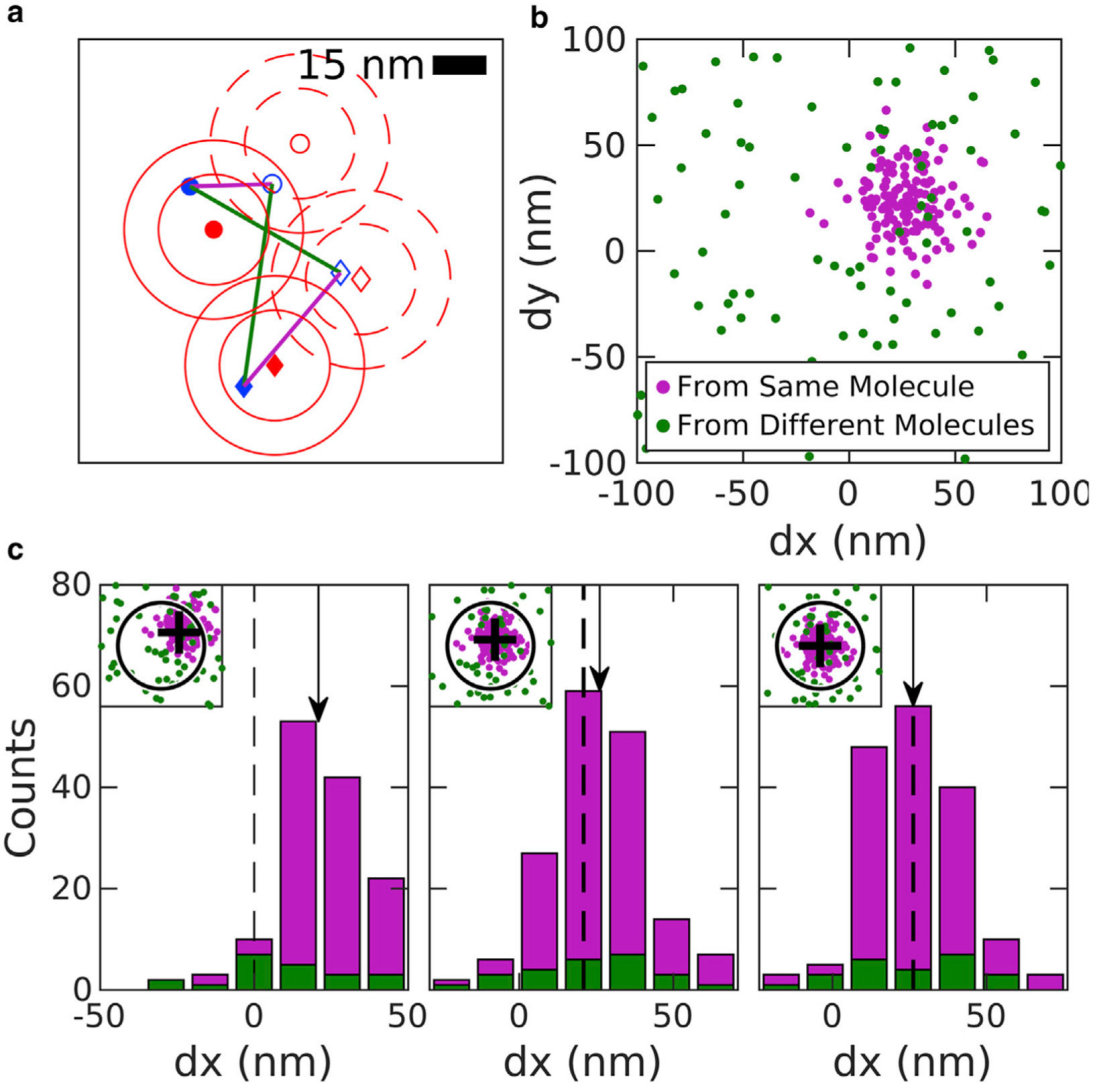
Demonstration of the MS algorithm. (*a*) A sample 2D image containing two molecules (filled *red symbols*) that are each localized one time (filled *blue symbols*). At a later time, the molecules are translated 25 nm in both dimensions (*open red symbols*) and two additional localizations are acquired (*open blue symbols*). Red contours indicate one and two times the localization precision around molecule centers. Straight lines show displacements between localizations acquired at distinct times. Some connect localizations from the same displaced molecule (*magenta*), whereas others connect different molecules (*green*). (*b*) Displacements like those shown in (*a*) displayed as points. Points connecting the same molecules cluster around the displacement, whereas points connecting different molecules produce a uniform background. (*c*) Three iterations of the MS algorithm showing the displacements as histograms in one dimension and as points in 2D in the inset. (*Left*) Initially, a region of interest (*circle in inset*) is centered at zero shift. The mean displacement of this subset of points is found (*arrow in main graph* and *cross in inset*). (*Middle*) A new region of interest is drawn around the mean from the initial iteration. The mean displacement from this subset of points (*arrow*) is shifted to slightly more positive values than the previous mean. (*Right*) At the final iteration, the tabulated mean (*arrow*) is equivalent to the starting point (*dashed line*) because the peak is centered within the region of interest.

A graphical illustration of the MS algorithm as applied to sample 2D localizations is presented in Fig. 1. The localizations all lie in one of two data sets which sample the same uniformly distributed emitters, but with a constant relative shift **r**_shift_ in space. The first step of the algorithm is to extract pairwise displacements between all localizations across the two data sets. When individual displacements are plotted as points (Fig. 1 b), displacements arising from the same labeled objects (magenta points) cluster around **r**_shift_, whereas displacements arising from different objects (green points) distribute randomly over space. The MS algorithm determines the center of the peak of the distribution through iteration (27-29). At each iteration, all pairs within the radius of consideration are extracted, and the updated shift estimate is the centroid of these pairs. The uniformly distributed background will tend to bias the centroid toward the center of the observation window, whereas the peak moves the mean toward **r**_shift_. The observation window is then redrawn around the new mean and the process is repeated until the peak is centered in the observation window. Three iterations of the algorithm are visualized in Fig. 1 c.

Although the emitters of Fig. 1 are distributed uniformly in space, leading to the uniform distribution of the pairs from different emitters, the MS method does not depend on this assumption. In samples in which emitters are organized into structures or randomly clustered, the pairs arising from different emitters are also more likely to be at shorter distances, so that the distribution of green points in Fig. 1 b will also be peaked at **r**_shift_. However, in our experience, pairs of localizations from the same emitter are more important for the MS and other drift estimates. We also note that our analysis assumes that emitters that are localized in one data set remain within the field of view in the second data set, and vice versa. This may not always be the case, and could in principle lead to bias in shift estimates, but in practice this is typically a negligible effect. Roughly, the contribution of fluorophores near the edge of the field of view may be biased by up to about the localization precision, and the fraction of fluorophores that are affected is restricted to those that lie within about a localization precision from the edge of the field of view, in the direction of the drift. So, for example, in a 100 *μ*m field of view with localization precision of 15 nm, we would expect this bias to be on the order of a picometer.

**FIGURE 2:**
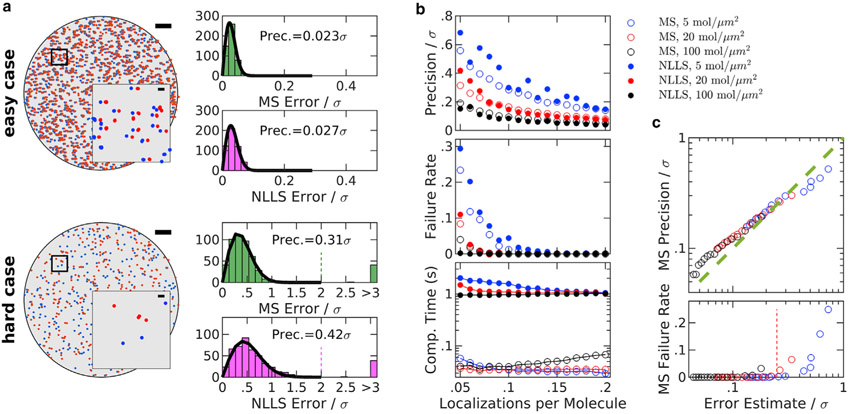
Evaluating the MS algorithm on simulated data translated in two dimensions, compared with nonlinear least-squares (NLLS) fitting approach. Simulations and displacement determination approaches are described in Supporting methods and materials. (*a*) Two examples of simulated data sets. Scatter plots on the left show a representative configuration for an extremely easy case, 5 mol/*μ*m^2^, with an average of two localizations per molecule (*top*) and a relatively hard case 20 mol/*μ*m^2^, with an average of 0.05 localizations per molecule (*bottom*). Scale bars, 2 *μ*m (*large image*) and 150 nm (*inset*). The simulated localization precision is *σ* = 15nm. Histograms on the right show errors evaluated for the MS and NLLS approaches for the two cases considered, evaluated from 500 simulations with random displacements between 0 and 150 nm (10*σ*). The precision of each method (Prec.) is evaluated as the standard deviation (SD) of a Gaussian fit to the central peak of the histogram (*solid line*). The “failure rate” is the fraction of simulations for which the error exceeds twice the localization precision *σ*, indicated as a dashed line for the hard case. (*b*) Performance comparison of the MS and NLLS approaches over a broad range of simulated conditions. Each point summarizes 500 simulations of the indicated average density and localizations per molecule, with precision and failure rate evaluated as described in (*a*), along with the average computation time per calculation. (*c*) The measured precision plotted as a function of an error in the displacement estimated from data, as derived in Supporting methods and materials. MS failure rate versus error estimate shows that the MS algorithm loses robustness when the estimated error exceeds *σ*/4 (*dashed line*).

To benchmark this MS approach, we evaluated the ability of the algorithm to detect known shifts of simulated data sets of a circular test cell, as summarized in Fig. 2. Shifts were estimated by both the MS algorithm and by NLLS fitting of a Gaussian to the spatial cross-correlation function of the two data sets, as implemented in the supporting software provided with (21). The performance of each algorithm was similar for easy cases that produce a well-defined peak at **r**_shift_. An extremely easy case is depicted at the top of Fig. 2 a, which single molecules are well spaced (surface density = 5/*μ*m^2^) and their positions are well sampled in both frames (twice per molecule on average). In this case, the shift can be clearly identified by eye, and both algorithms reliably and accurately identify the displacement between frames. The simulation depicted at the bottom of Fig. 2 a represents a much harder case, in which molecules are present at higher surface density (20/*μ*m^2^) and only 1 in 20 molecules are imaged on average in a given data set. In this case, MS modestly outperforms NLLS fitting, both by locating the peak with improved precision and by more reliably finding the peak overall. These trends hold over simulations conducted over a broad range of molecular densities and localizations per molecule (Fig. 2 b). We also estimated shifts from the overall center of mass of each data set, which yielded precisions more than an order of magnitude worse than both the MS and NLLS methods. Moreover, MS is more computationally efficient than NLLS, largely because fast Fourier transforms are not computed in the MS approach. This improvement in speed is enabled through the use of a particularly efficient algorithm from the R package spatstat (30) to extract pairwise displacements between nearby points (see Supporting methods and materials).

For large displacements, both the MS and NLLS algorithms applied in Fig. 2 require an initial step to identify an approximate starting point for the higher accuracy calculation. Fig. S1 shows the failure rate of each algorithm as a function of the distance of the start point from the true shift. MS robustly identifies the main peak over a broad range of simulation conditions as long as it resides within the initial observation window, so large shifts can be identified simply using a large window in the first iteration. This window is typically 100 nm for experimental localizations and 150 nm for the simulations of Fig. 2. NLLS robustly identifies the main peak when the starting point for the computation falls within the localization precision of the peak of the cross-correlation function. In many practical cases, the peak is much farther from the origin than the localization precision, so a separate method is needed to identify a suitable starting point. Here, this is accomplished using a particularly effective algorithm that identifies the global maximum in a smoothed cross-correlation function, as described in the supporting material of (21). The robustness of the NLLS fitting approach is dependent on the ability of this algorithm to identify a suitable starting point over a broad range of simulation conditions. Note that data sets for which the emitter distribution is highly structured or clustered typically lead to improved performance of the start point identification routine by introducing a broad peak in the cross-correlation function in addition to the sharp peak that represents repeat localizations of the same fluorophore.

This MS approach is applied to SMLM localizations that experience continuous drift by distributing localizations into nonoverlapping temporal bins with equal numbers of frames, and displacement estimates are tabulated between all possible pairs of bins. The number of frames in each temporal bin is an important parameter; short temporal bins have few localizations per molecule, so individual displacements may be estimated imprecisely. Long temporal bins have more localizations per molecule and more precise drift estimates but reduce the time resolution of the drift estimate. A linear least-squares fitting algorithm is then used to generate a trajectory that passes through control points positioned, at times, centered on each temporal bin, as described previously (21), taking advantage of the high redundancy to improve precision of the control points. We have slightly modified this past approach by including weights in the linear least-squares fitting, where weights are determined directly from data using a relation that approximates error in the mean displacement (described in Supporting methods and materials), as demonstrated in simulated data sets (Fig. 2 c). Briefly, errors are reduced when there are more pairs originating from the same molecules (*magenta points* in Fig. 1) and errors increase when more pairs originate from different molecules within the observation window (*green points* in Fig. 1). Estimated errors can also act as a proxy for overall reliability of the algorithm. Fig. 2 c also shows that MS reliably finds the desired peak when the estimated error remains smaller than one-quarter of the localization precision. This observation can act as a guide when selecting the number of frames included in temporal bins.

It is tempting to distribute frames into overlapping temporal bins, which in principle could improve time resolution while retaining a sufficient number of localizations to accurately determine displacements. However, we find that drift estimates from overlapping time bins are subject to substantial bias, underestimating the actual displacements accrued over time (Fig. S2 a). This occurs because the same localizations are present in adjacent bins, biasing the result toward **r**_shift_ = 0. Similar bias can arise even in the absence of overlapping time bins because SMLM data frequently contain time-correlated localizations arising from the finite off rates of fluorescent blinking (PALM/dSTORM) or binding (PAINT). These factors mean that pairs of localizations from the same fluorophore are mostly from time separations that are shorter than the time difference between the bin centers and therefore underestimate the average drift between the bins.

**FIGURE 3:**
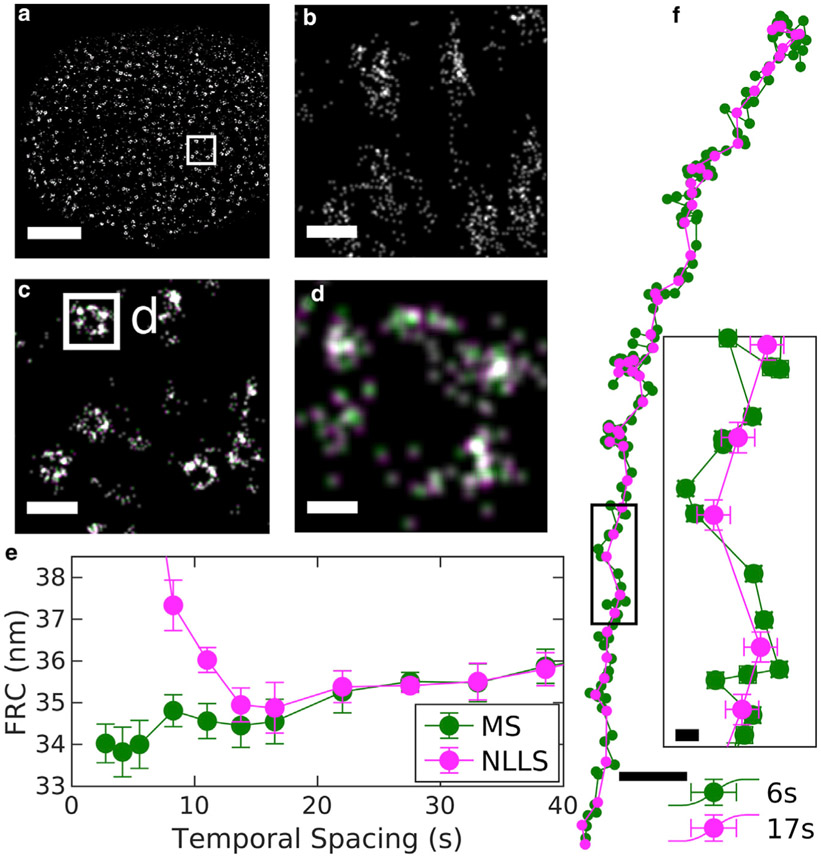
Demonstration of MS drift correction of a 2D SMLM data set of antibody-labeled Nup210 in nuclear pore complexes (NPC) within the nuclear envelope of primary mouse neurons. This data set contained 15,500 image frames acquired over 14 min, with an average localization precision of 8 nm. (*a*) Reconstructed image of a single nucleus that is a subset of this data set. (*b*) Reconstruction without drift correction of the region shown within the white box in (*a*). (*c*) Same region as in (*b*) but with drift correction. (*d*) An image of a single complex demonstrates the modest shifts in localizations due to drift corrections estimated with MS and 6 s (*green*) or NLLS and 17 s (*magenta*) temporal bins. Scale bars, 2 *μ*m (*a*), 200 nm (*b* and *c*), and 30 nm (*d*). (*e*) Fourier ring correlation (FRC) resolution after applying drift corrections estimated using the specified temporal bin widths for the MS and NLLS methods. Error bars represent the SD over 20 replicates of the FRC calculation. (*f*) Estimated drift trajectories evaluated from using the method and temporal spacing specified. Error bars represent 68% confidence intervals from the weighted least-squares drift estimation of each control point. Scale bars, 20 nm for the overall drift curve and 2 nm in the inset.

The MS approach is applied to a 2D experimental data set of nuclear pore complexes (NPCs) in Fig. 3. NPC assemblies are labeled with primary and secondary antibodies against Nup210 within the nuclear envelope of intact primary mouse neurons, and Fig. 3, a-d shows reconstructed images at various magnifications. Fig. 3 b is a reconstruction produced without drift correction, in which localizations from single NPCs are smeared over a large area, highlighting the importance of drift correction.

The performance of the MS algorithm was tested on this data set by generating multiple drift trajectories through binning with different temporal resolutions. These trajectories were each applied to the full SMLM data set, and Fourier ring correlation (FRC) (31,32) was used to quantify image resolution (Fig. 3 e). For comparison, we conducted drift corrections using the redundant cross-correlation NLLS approach, as described previously (21). In this case, MS modestly outperforms NLLS fitting, allowing for accurate drift correction with smaller temporal bins and modestly improving the resolution of the reconstructed image. We used this data set to explore possible bias introduced because of temporal correlations of single-fluorophore blinking by running the linear least-squares algorithm and including or excluding adjacent pairs of bins on the data of Fig. 2. We found no significant difference between the two cases (Fig. S2 b), indicating that the impact of this bias is negligible within experimental errors. Additional diagnostics for the MS and NLLS approaches are shown in Fig. S3.

Drift trajectories are shown in Fig. 3 f for temporal bin widths that produce accurate FRC metrics for the MS and NLLS approaches. For MS, a temporal bin slightly larger than the minimum from the FRC curve is used because this produces smaller errors on individual control points. As expected, the drift trajectories follow the same general shape, but the trajectory generated from MS has improved time resolution. In parts of the trajectory, the errors of the control points are smaller than the distance between the trajectories. In these regions, higher time resolution yields improved spatial resolution in the final reconstructed image. Although the differences in the trajectories are significant, their impact is not apparent when viewing reconstructed images of entire nuclei or collections of NPCs, as in Fig. 3, a and c. Differences become more apparent in images of individual pores, in which displacements of several nanometers shift the relative positions of labeled subunits (Fig. 3 d).

The MS algorithm is easily extended to localizations acquired in 3D, in which performance improvements are more evident compared with the established NLLS approach. Because the MS algorithm uses points instead of reconstructed images and fast Fourier transforms, it can be extended into 3D without needing expanded memory resources that limit the practical application of NLLS in 3D. Instead, the 3D application of NLLS drift correction is typically accomplished by generating 2D projections that contain less information than the 3D localizations from which they are produced (21). To see why, consider a pair of emitters that are close together in x-y but far apart in z. Pairs of localizations from this pair of fluorophores will be included in 2D MS drift estimation when using data projected into the *x-y* plane but excluded from the full 3D drift estimation method by virtue of their large separation in *z*. We compare the precision and robustness of 3D MS and NLLS on simulated localizations spread over a cylindrical volume in Fig. S4, in which the NLLS correction is performed on projections into the *x-y*, *x-z*, and *y-z* planes, as described in (21). We also directly compare the *x-y* performance of the full 3D MS method with the 2D MS method performed on data projected into the *x-y* plane (Fig. S5).

**FIGURE 4:**
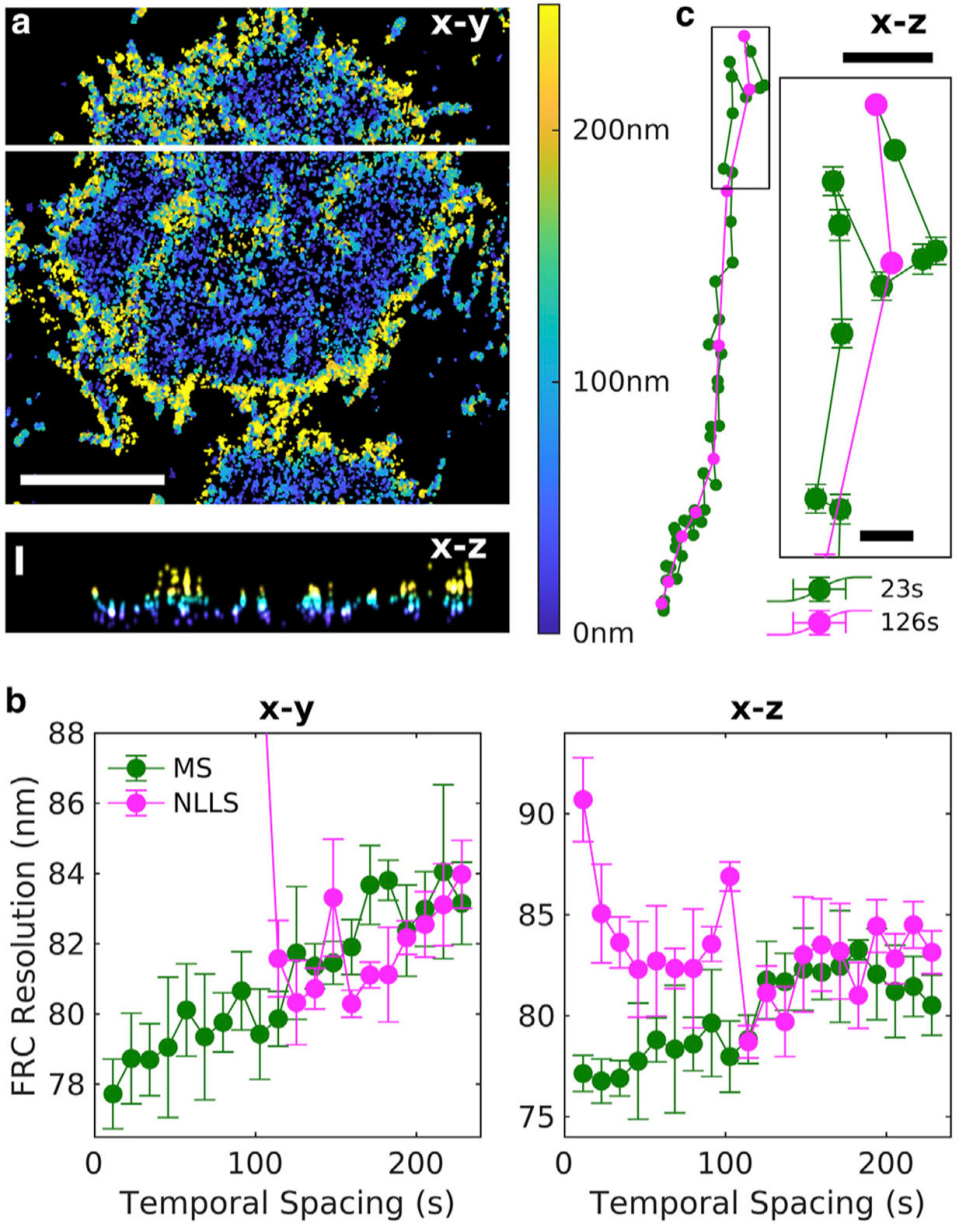
Demonstration of MS drift correction of a 3D SMLM data set of B cell receptors at the ventral plasma membrane of CH27 B cells. This data set contained more than 400,000 localizations acquired over 15 min, with an average localization precision of 17 nm in the lateral (*x-y*) dimension and 31 nm in the axial (*z*) dimension. (*a*) Reconstructed image of a subset of this data set showing the average z position within each *x-y* pixel, as indicated in the color bar. *x-z* slice at the position drawn as a white line is shown below. Scale bars, 5 *μ*m for *x-y* and 200 nm for *z*. (*b*) Fourier ring correlation (FRC) estimates of image resolution after applying drift corrections estimated using the specified temporal bin widths for the MS and NLLS methods. Error bars represent the SD over five replicates of the FRC calculation. (*c*) Estimated drift trajectories evaluated with the specified temporal spacing. Error bars represent 68% confidence intervals from the weighted least-squares drift estimation of each control point. Scale bars, 50 nm (10 nm in the inset).

Fig. 4 applies the MS approach to an experimental SMLM data set of labeled B cell receptors on the ventral membrane of B cells imaged using a phase mask in the emission path to localize fluorophores in 3D (8). As was the case for simulated data sets, the differences in the performance of the MS and NLLS fitting methods are more pronounced than in the 2D data set of Fig. 3. Additional diagnostics for the 3D case are shown in Fig. S6.

In summary, a mathematically simple MS algorithm modestly outperforms cross-correlation-based estimates of drift correction in 2D and more significantly improves the time resolution of drift corrections in 3D. The approach is computationally efficient, is robust without sophisticated methods to estimate starting points, and does not require image reconstruction with memory and pixelation limitations. The metric provided to estimate error and predict robustness directly from data provides users with a means to evaluate the quality of a drift correction within an SMLM analysis pipeline. For the example data sets explored, modest improvements in resolution lead to adjustments of localized molecule positions relevant for evaluating the structure of protein complexes in cells.

## Supplementary Material

1

## WHY IT MATTERS

Drift correction is a regular part of the analysis pipeline in super-resolution fluorescence localization microscopy because the microscope stage typically moves farther than the localization precision over the time needed to acquire an image. This work presents a mathematically simple mean shift (MS) algorithm that allows for accurate drift correction with high time resolution from acquired localizations in two or three dimensions.
