# Supplementary material for: A mean shift algorithm for drift correction in localization microscopy
Source: Biophys Rep (N Y). 2021 Jul 24;1(1):100008. doi: 10.1016/j.bpr.2021.100008 (PMC8978553; doi:10.1016/j.bpr.2021.100008)
Supplement: Document S1. Supporting materials and methods and Figs. S1–S6 [file mmc1.pdf]

Biophysical Reports, Volume *1*

Supplemental information

A mean shift algorithm for drift correction in localization microscopy

Frank J. Fazekas, Thomas R. Shaw, Sumin Kim, Ryan A. Bogucki, and Sarah L. Veatch

Supplemental Methods and Materials

Simulated Datasets

An idealized 2D SMLM dataset was simulated as a spatially random set of fluorophores on a 20μm diameter circular cell, with each fluorophore giving rise to a Poisson-distributed number of localizations with isotropic Gaussian localization error $\sigma_x = \sigma_y = 15/\sqrt{2}$ nm. We define $\sigma = \sqrt{\sigma_x^2 + \sigma_y^2} = 15$ to denote the total root-mean-square localization error. A second dataset was generated from the same fluorophore locations, localization precision, and average number of localizations per fluorophore, and shifted between 0 and 150 nm in a random direction. Simulated datasets were generated over a range of densities (5 to 100 per μm^2) and a range of localizations per molecule (.05 to .2).

An idealized 3D SMLM dataset was simulated in a similar fashion. Fluorophores were distributed uniformly on a cylinder 20 μm in diameter and 2 μm deep. Each fluorophore produces a Poisson-distributed number of localizations with $\sigma_x = \sigma_y = 15/\sqrt{2}$ nm as before, and with $\sigma_z = 30/\sqrt{2}$ nm. One dataset is translated by a random distance between 0 and 150 nm in a random direction in x, y, and z.

Extracting Close Pairs of Coordinates Between Datasets

Consider two point sets $\mathbf{u}_i = (u_{ix}, u_{iy})$ and $\mathbf{v}_j = (v_{jx}, v_{jy})$, for $i = 1, ..., n_1$ and $j = 1, ..., n_2$. We wish to quickly determine which pairs (i, j) are closer than some maximum distance $r_{\max}$; i.e. which pairs satisfy $\|\mathbf{u}_i - \mathbf{v}_j\| < r_{\max}$. The algorithm is adapted from the code for the `closepairs()` and `crosspairs()` functions of the R package spatstat (1), and implemented in C with a MATLAB interface. We first sort each dataset with respect to its x-coordinate, so that $u_{kx} \le u_{lx}$ whenever $k \le l$. Then the algorithm proceeds as follows:

1. Let $i = 1$ and $j_{\text{left}} = 1$.
2. Let $x_{\text{left}} = u_{ix} - r_{\max}$. All close pairs of $\mathbf{u}_i$ must satisfy $v_{jx} > x_{\text{left}}$.
3. Increment j_{left} until $v_{j_{\text{left}}x} \ge x_{\text{left}}$.

4. For each $j = j_{left},\dots,n_2$, if $v_{jx} - u_{ix} > r_{\max}$, increment i and return to step 2. Otherwise, compute $r_{ij}^2 = (u_{ix} - v_{ix})^2 + (u_{iy} - v_{iy})^2$. If $r_{ij}^2 \le r_{\max}^2$, add (i, j) to the list of results.

This algorithm avoids computing pairwise distances between most pairs in the dataset, and so is much faster and more memory efficient than a brute force approach. It can be readily adapted to higher dimensions by applying the appropriate *n*-dimensional distance metric in step 4. For convenience, our implementation returns the displacements $\Delta \mathbf{r}_{ij} = \mathbf{v}_j - \mathbf{u}_i$, and total distance $r_{ij} = \|\Delta \mathbf{r}_{ij}\|$ for each pair (i, j), instead of the indices themselves.

Determining Shifts between Translated Datasets Using a Mean Shift Algorithm

Given the set of displacements $\Delta \mathbf{r}_{ij} = \mathbf{u}_i - \mathbf{v}_j$ between two point sets $\mathbf{u}_i$ and $\mathbf{v}_j$, a mean shift clustering algorithm (2–4) can be applied to search for the peak of the displacement density function. Briefly, let $\mathbf{r}_{\text{shift},0}$ be an initial guess to initialize the shift estimate, and δ a radius of consideration to use in the optimization procedure. The algorithm proceeds by iteration, by setting

$$\mathbf{r}_{\text{shift},t+1} = \left\langle \mathbf{r}_{ij} \right\rangle_{\left\|\mathbf{r}_{ij} - \mathbf{r}_{\text{shift},t}\right\| \le \delta},$$

where the average is restricted to the subset of displacements $\mathbf{r}_{ij}$ that satisfy the subscript, i.e. that are within a radius δ from the previous shift estimate $\mathbf{r}_{\text{shift},t}$. The algorithm terminates when the distance $\left\|\mathbf{r}_{\text{shift},t+1} - \mathbf{r}_{\text{shift},t}\right\|$ between subsequent shift estimates becomes smaller than machine precision, or when the number of iterations exceeds a user-defined maximum number. δ must be sufficiently large so that the true shift resides within the explored area when centered at the starting-point. In practice, we apply the algorithm twice: first with a large δ to determine the rough shift, and then with a smaller δ, using the first estimate as a starting point, to refine the estimate.

While the above can be applied directly to 3-dimensional data by taking the average over a 3-dimensional ball of radius δ instead of the 2-dimensional disc, we find it is advisable to consider an ellipsoid that is stretched in the z-direction, to account for the larger axial localization errors present in our 3-dimensional simulated and experimental datasets. In the present work, we let the semimajor axis of the ellipsoid be $\sqrt{2}\delta$, in the z direction, and the semiminor axes both δ, so that x-y cross-sections of the regions of consideration are discs.

Estimates of mean shift error

We model the distribution of pairs around the true shift as a Gaussian-distributed peak with standard deviation ς, centered on the true shift $\mathbf{r}_{\text{shift}}$ on a uniformly distributed background. Assuming $\mathbf{r}_{\text{shift},t}$ is sufficiently close to $\mathbf{r}_{\text{shift}}$ that most of the Gaussian peak falls within the region of consideration, the variance ξ^2 of the two components of $\mathbf{r}_{\text{shift},t}$ is given by

$$\xi^2 = \mathrm{Var}\left[r_{\text{shift},t,x}\right] = \mathrm{Var}\left[r_{\text{shift},t,y}\right] = \frac{n_{\text{true}}\varsigma^2 + n_{\text{false}}\delta^2/4}{(n_{\text{true}} + n_{\text{false}})^2},$$

where δ is the radius of consideration for the MS algorithm, and n_{true} and n_{false} are respectively the number of "true pairs" that are drawn from the Gaussian part of the distribution (displacements between different localizations of the same molecules) and the number of "false pairs" that are drawn from the uniform part (displacements between different molecules), that fall within the region of consideration. Furthermore, the expected value after one more step can be derived:

$$\begin{aligned}\mathrm{E}\left[\mathbf{r}_{\text{shift}.t+1} - \mathbf{r}_{\text{shift},t}\right] &= \frac{n_{\text{false}}\mathbf{r}_{\text{shift}.t} + n_{\text{true}}\mathbf{r}_{\text{shift}}}{n_{\text{true}} + n_{\text{false}}} - \mathbf{r}_{\text{shift,t}} \\ &= \frac{n_{\text{true}}}{n_{\text{true}} + n_{\text{false}}}\left(\mathbf{r}_{\text{shift}} - \mathbf{r}_{\text{shift},t}\right).\end{aligned}$$

Suppose t is the final step of the algorithm, i.e. $\mathbf{r}_{\text{shift},t+1} - \mathbf{r}_{\text{shift},t} = 0$. Then by hypothesis, $\mathbf{r}_{\text{shift},t}$ deviates from its expected value by

$$\frac{n_{\text{true}}}{n_{\text{true}} + n_{\text{false}}}\left\|\mathbf{r}_{\text{shift},t} - \mathbf{r}_{\text{shift}}\right\|.$$

This deviation will typically take on values comparable to the standard deviation ξ shown above. Thus, we estimate the error of the MS algorithm by:

$$\text{Predicted Error} = \frac{\sqrt{n_{\text{true}}\varsigma^2 + n_{\text{false}}\delta^2/4}}{n_{\text{true}}}.$$

This predicted error is to be interpreted as an estimate of the standard deviation of the shift estimate in each direction.

In practice, the parameters n_{true}, n_{false}, and ς are not known, so we estimate them from data. Specifically, we construct the isotropic cross-correlation function $c(r)$ from the pair separations $\mathbf{r}_{ij}$, determine the baseline of $c(r)$ from its long-range median value, and use the baseline to infer n_{true} and n_{false}. Finally, we fit $c(r)$ to a Gaussian plus a constant to estimate ς.This error estimate is derived from a heuristic argument and is not exact. However, its performance is adequate in practice. See Figure 2c for a comparison to observed standard deviations of MS shift estimates.

For 3D data, we compute lateral and axial predicted errors separately, by projecting the data from the ellipsoidal region of consideration into the x-y plane or onto the z axis, respectively. n_{true}, n_{false}, and ς are estimated separately for the lateral and axial directions from the two projections.

Evaluating displacements using nonlinear least squares (NLLS) fitting

Displacements $\mathbf{r}_{\text{shift}}$ between pairs of localization datasets were also estimated by NLLS fitting of a Gaussian to the spatial cross-correlation function of the two datasets. NLLS fitting was accomplished using software published as Supplemental material of (5). Images were first reconstructed from simulated localizations with a pixel size of 15nm for simulated localizations, or from acquired data with a pixel size of 8nm for Nup210 or 15nm for B cell receptor experimental localizations. Cross-correlations are tabulated using 2D Fast Fourier Transforms (FFTs) and then fit a 2D Gaussian function to a subset of the cross-correlation centered at the start-point of the NLLS algorithm. The software from (5) finds the start-point using an elegant smoothing step to reduce noise then uses the largest local maximum of the smoothed cross-correlation as the start-point for fitting.

For localizations acquired in 3D, multiple 2D projections were constructed from 3D localizations, then the procedures described for 2D images were applied to determine displacements. First, images projecting on the lateral dimension (x-y plane) were generated and the lateral displacement was determined. To compute the z displacement, both the xz and yz

projections were used, and the final z displacement was the average determined from the two projections.

Correcting continuous drift

Continuous drift was corrected by temporally dividing the data into N bins, each having the same number of frames. For each of the $N(N-1)/2$ pairs (m,n) of temporal bins, the mean shift or NLLS algorithm is applied to estimate the shift $\mathbf{r}_{\text{shift},m\rightarrow n}$ from temporal bin m to n, corresponding to the drift between the bins. Drift at each of the N time points is calculated from the $N\,(N-1)/2$ pairwise shifts using a least-squares minimization algorithm (5); this takes advantage of the overdetermined nature of the drift calculation to improve the precision of the measurement. Outlier shifts, whose residual with respect to the least-squares estimate exceeds a user-defined threshold, can also be discarded as described in (5). These shifts typically correspond to "failures" of the shift estimation method. The final drift curve at each frame is determined by linear interpolation and extrapolation from the N basis points.

Evaluating performance of displacement algorithms

For simulated localizations, errors away from known displacements were tabulated for each simulated configuration. The 2D precision of each method is defined as the standard deviation of a centered, isotropic 2D Gaussian fit to the central peak of the histogram of these values, considering only values that fall within twice the localization precision (2σ) used in the simulation. The fit is applied directly to the absolute errors $\left\|\mathbf{r}_{\text{shift,est.}}-\mathbf{r}_{\text{shift}}\right\|$ with the distribution function $f(r)=\frac{r}{\sigma^2}e^{-r^2/2\sigma^2}$. Similarly, in the 3D case, x- and z-precision are evaluated separately by fitting 1D Gaussian functions to the x- and z-errors, respectively. Values that fall outside of the 2σ window are reported as failures of the algorithm, and contribute to the failure rates reported in figures. Since failures can return values with large errors, they can have an outsized impact on simpler precision metrics, such as the root mean square error (RMSE). Computation time was assessed in MATLAB using the built-in `tic` and `toc` functions. For simulated data, computation time was averaged over 500 simulations for each condition. For experimental data, computation time includes the $N(N-1)/2$ shift estimates and the error estimates for each shift

estimate in the MS case. Normalized residual degrees of freedom (Normalized DOF) of the linear least squares algorithm are calculated by the ratio of shifts that are used for the final least squares minimization step (after removal of outliers) to the number of time points at which drift is estimated (i.e. $N-1$). This serves as a diagnostic for how much redundancy is included in the linear least squares minimization step.

Evaluating the resolution of drift-corrected datasets

Resolutions of the final reconstructed images were compared using Fourier Ring Correlation (6, 7). Specifically, we used code adapted from the supplementary software of (6). To compute the x-y resolution, nearby localizations belonging to adjacent camera frames were grouped together, with the position taken to be the average of the relevant coordinates. The FRC curves were produced by dividing the dataset into blocks of 500 frames and allocating an equal number of blocks randomly to each of the two sets. The pixel size was taken to be 5nm. For the B cell dataset, the Fourier Ring approach was applied to the xy and xz projections in turn, also using a pixel size of 5nm in each case.

Preparation of cellular samples for imaging

Mouse primary neurons were isolated from P0 mouse pups that were decapitated and brains were isolated into ice cold, filtered dissection buffer (6.85 mM sodium chloride, 0.27mM potassium chloride, 0.0085mM sodium phosphate dibasic anhydrous, 0.011mM potassium phosphate monobasic anhydrous, 33.3mM D-glucose, 43.8mM sucrose, 0.277mM HEPES, pH 7.4) as described in (8). After removing the cerebellum and the meninges, cortices were dissected out, placed into a microcentrifuge tube, and cut into small pieces with dissection forceps. Cortices were incubated in 50µL papain (2mg/mL; BrainBits) and 10µL DNase I (1mg/mL; Worthington Biochemical) for 30min at 37 °C. 500µL BrainPhys Neuronal Medium (Stemcell Technologies) and 10µL additional DNase I were added, and cortices were titurated using P1000 and P200 pipet tips. Titurated cortices were centrifuged at 1000rpm for 5min. After discarding the supernatants, the pellets were titurated and centrifuged three more times until the supernatant remained clear and neuronal pellets were visible. Pelleted neurons were resuspended in BrainPhys Neuronal medium with SM1 supplement as previously described (9), then plated onto 35mm #1.5 glass-bottom dishes (MatTek Life Sciences) coated with polyethlenimine

(100 μg/ml; Polysciences). Neurons were incubated in 5% CO_2 at 37 °C, and 1mL of media was replaced every four days.

On day 10 of culture (days *in vitro* 10), neurons were rinsed with sterile Hank's Balanced Salt Solution, then incubated for 1min with pre-warmed 2% PFA (Electron Microscopy Sciences) in Phosphate Buffered Saline (PBS). The neurons were then incubated in 0.4% Triton X-100 (Millipore Sigma) in PBS for 3min, and fixed for 30min with 2% PFA in PBS. Neurons were then washed with PBS five times, incubated in blocking buffer containing 5% Normal Donkey Serum and 5% Normal Goat Serum (Jackson Laboratories) for 30min, then labeled with Nup210 polyclonal antibody diluted in blocking buffer (1:200; Bethyl laboratories A301-795A) overnight in 4 °C. The following day, neurons were washed three times in PBS then stained with Goat-anti-rabbit Alexafluor 647 secondary antibody (1:1000; Thermo Fisher) for an hour, washed three times with PBS, then imaged.

CH27 B cells (10) were cultured, allowed to adhere to 35mm #1.5 glass-bottom dishes (MatTek Life Sciences) overnight, then incubated in Alexa647 conjugated fAb prior to fixation in 4% PFA and 0.1% gluteraldehide (Electron Microscopy Sciences), as described previously (11). The labeled fAb antibody was prepared by conjugating an Alexa647 NHS ester (ThermoFisher) to an unconjugated fAb (Goat Anti-Mouse IgM, μ chain specific; Jackson Immunoresearch) using established protocols (11).

Single molecule imaging and localization

Imaging was performed using an Olympus IX83-XDC inverted microscope. TIRF laser angles where achieved using a 60X UAPO TIRF objective (NA = 1.49), and active Z-drift correction (ZDC) (Olympus America) as described previously. The ZDC was not used for collection of 3D datasets. Alexa 647 was excited using a 647 nm solid state laser (OBIS, 150 mW, Coherent) coupled in free-space through the back aperture of the microscope. Fluorescence emission was detected on an EMCCD camera (Ultra 897, Andor) after passing through a 2x expander. Imaging in 3D was accomplished using a SPINDLE module equipped with a DH-1 phase mask (DoubleHelix LLC).

Single molecule positions were localized in individual image frames using custom software written in Matlab. Peaks were segmented using a standard wavelet algorithm (12) and segmented peaks were then fit on GPUs using previously described algorithms for 2D (13) or 3D localizations (14). After localization, points were culled to remove outliers prior to drift correction. Images were rendered by generating 2D histograms from localizations followed by convolution with a Gaussian for display purposes.

Supplemental Software:

Supplemental Software can be found at https://github.com/VeatchLab/Mean-Shift-Drift-Correction. The software contains Matlab and C code to run mean shift drift corrections on 2D and 3D SMLM data. We also include slightly modified versions of the NLLS and FRC codes published previously (5, 6). Three example scripts are also included:

1. meanshift_example.m: determine the shift between a single pair of sets of localizations sampling the same structure at different times. The example uses data from the 2D nuclear pore complex (NPC) dataset of Fig 3.
2. example_NPC.m: correct 2D drift from the full NPC dataset of Fig 3.
3. example_Bcell.m: correct 3D drift from the full B cell dataset of Fig 4.

Supplemental Figures:

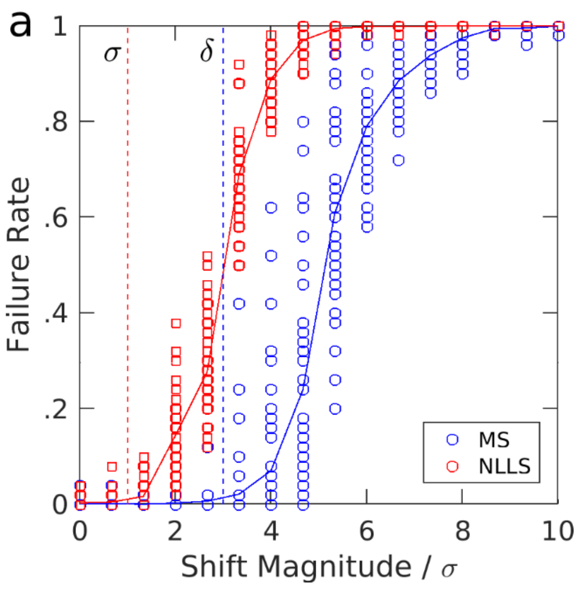

Figure S1. Evaluating the mean shift (MS) and NLLS algorithms with the start-point at the origin. Simulations and shift determination approaches are described in Methods. **a)** Shifts between 0 and 10 times the localization precision (σ) are applied in a random direction, and the MS and NLLS algorithms are applied to determine these displacements. The observation window (δ) for the MS algorithm has an extent of 3σ as indicated by the dashed blue line. The "failure rate" is the fraction of simulations whose error exceeds 2σ. Each point represents a given combination of fluorophore density and number of localizations per molecule, averaged over 50 independent trials. Densities range from 5 to 100 molecules per μm^2, and localizations per molecule range from .05 to .2.

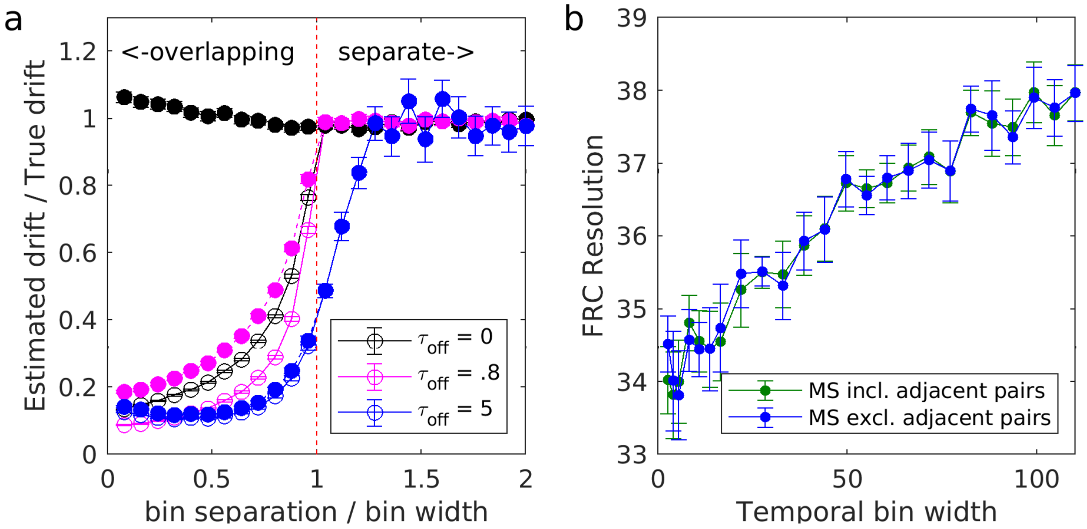

Figure S2. Drift estimates between overlapping temporal bins are prone to bias. a) Mean shift (MS) shift estimates for pairs of bins that are overlapping or separated by short times. Simulations are similar to those for Figure 2 with a cell of radius 10 µm and randomly distributed fluorophores at a density of 20 per µm^2, but with explicit blinking kinetics, modeled as a simple two state (fluorescent/dark) system, with activation and deactivation time constants τ_{on} and τ_{off} for the dark → fluorescent and fluorescent → dark transitions, respectively. A constant drift rate of .1 nm per frame is applied. The drift estimates shown here are for 50 frame temporal bins, with bin starts separated by the bin separation times as shown. For each τ_{off}, τ_{on} is adjusted so that the average number of localizations per fluorophore is approximately 0.15. Open circles represent drift estimates using all pairs of simulated points, including the trivial 0 displacements between points that appear in the overlap of the two temporal bins. Filled circles represent drift estimates using all pairs of distinct point, i.e. excluding the trivial 0 displacements between points that appear in the overlap of the two temporal bins. Note that overlapping bins are subject to substantial bias even for quite short τ_{off} (magenta points), and that even non-overlapping bins may be subject to bias when τ_{off} is long (blue points). b) FRC resolutions for the nuclear pore complex data of Figure 3, with MS drift corrections including or excluding drift estimates for adjacent pairs of temporal bins in the linear least squares fit.

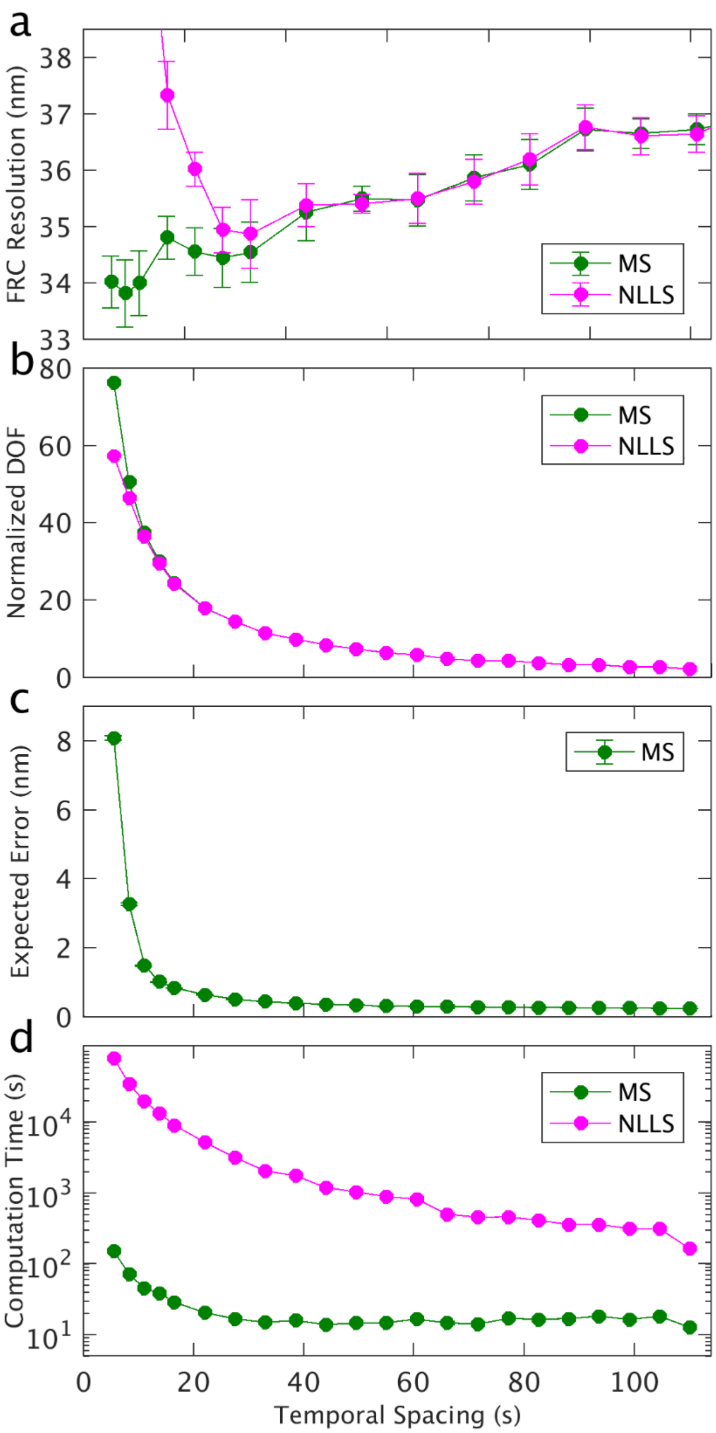

Figure S3. **Drift correction diagnostics for the nuclear pore complex dataset of Figure 3. a)** FRC resolutions. Error bars are given by the standard deviation over 20 trials. **b)** The number of degrees of freedom (DOF) after removing outliers (normalized by the number of parameters) for the redundant least square minimization calculation. **c)** RMSE of the expected errors for the mean shift method. Error bars are given by the standard error of the mean. **d)** Total computation time.

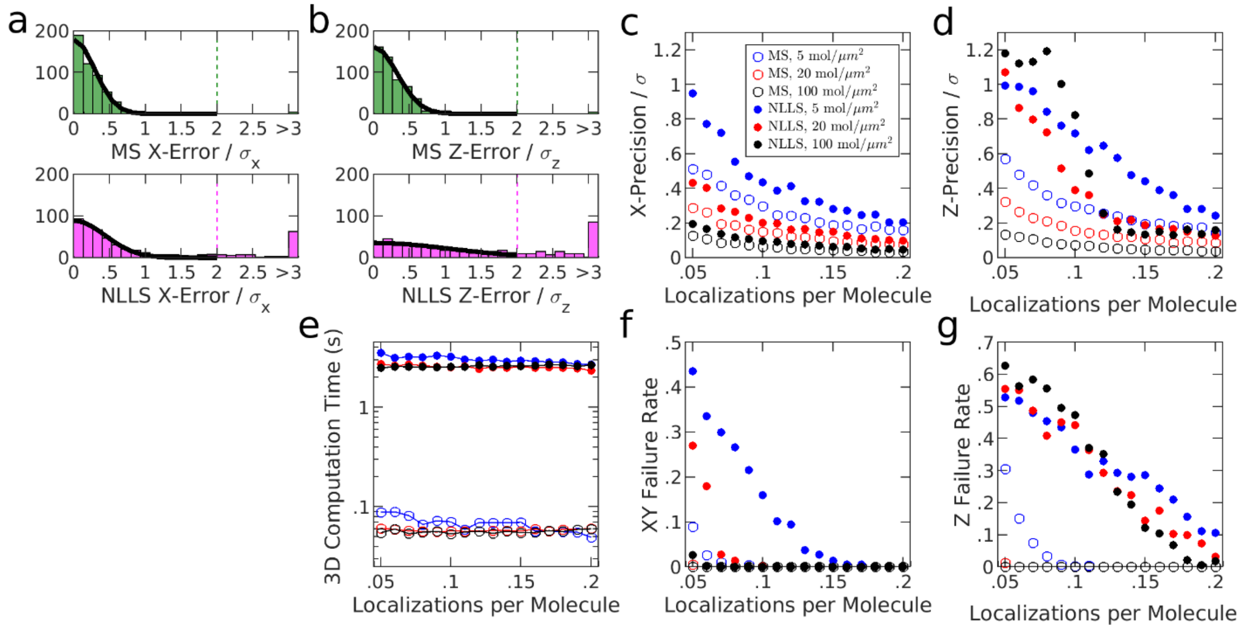

Figure S4. **Evaluating the mean shift (MS) algorithm on 3D simulated data, compared to the NLLS approach.** Simulations and shift determination approaches are described in methods. **a,b)** Histograms of x-errors **(a)** and z-errors **(b)** for the MS and NLLS approaches for the 0.01 molecules/µm^3 and 0.05 localizations per molecule condition. The precision of each method is evaluated for each condition as the standard deviation of a Gaussian fit to the central peak of the histogram. The "failure rate" is the fraction of simulations whose error exceeds twice the localization precision σ, indicated as a dashed line. Three densities are shown in the plots: 5. 20, and 100 molecules/µm^2. **c,d)** Comparison of the lateral **(c)** and axial **(d)** precision of each approach, plotted versus the number of localizations per molecule. **e)** Computation times of the two approaches over the same conditions shown in c,d). **f,g)** Comparison of the failure rate of each approach in lateral **(f)** and axial **(g)** directions under the same range of conditions.

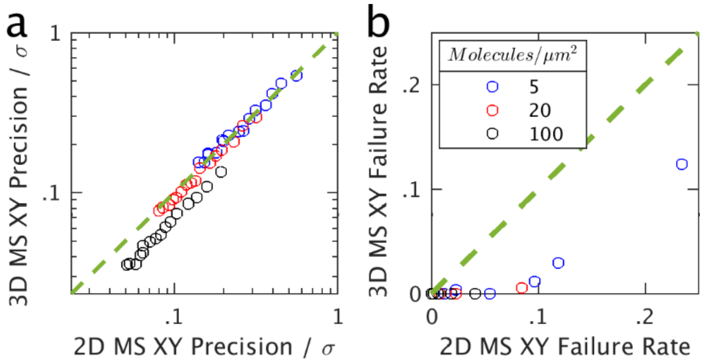

Figure S5. 2D projections of 3D data degrade mean shift (MS) shift estimation performance. Lateral (x-y) precision **(a)** and failure rate **(b)** when MS shift is determined in 3D or in 2D after projecting the localizations into the x-y plane. The points shown each summarize 500 replicates of one simulation condition, with fluorophore density as shown in the legend, and localizations per molecule ranging from .05 to .2. The simulated 3D data used here is constructed such that its 2D projection is identical to the 2D data used for Figure 2.

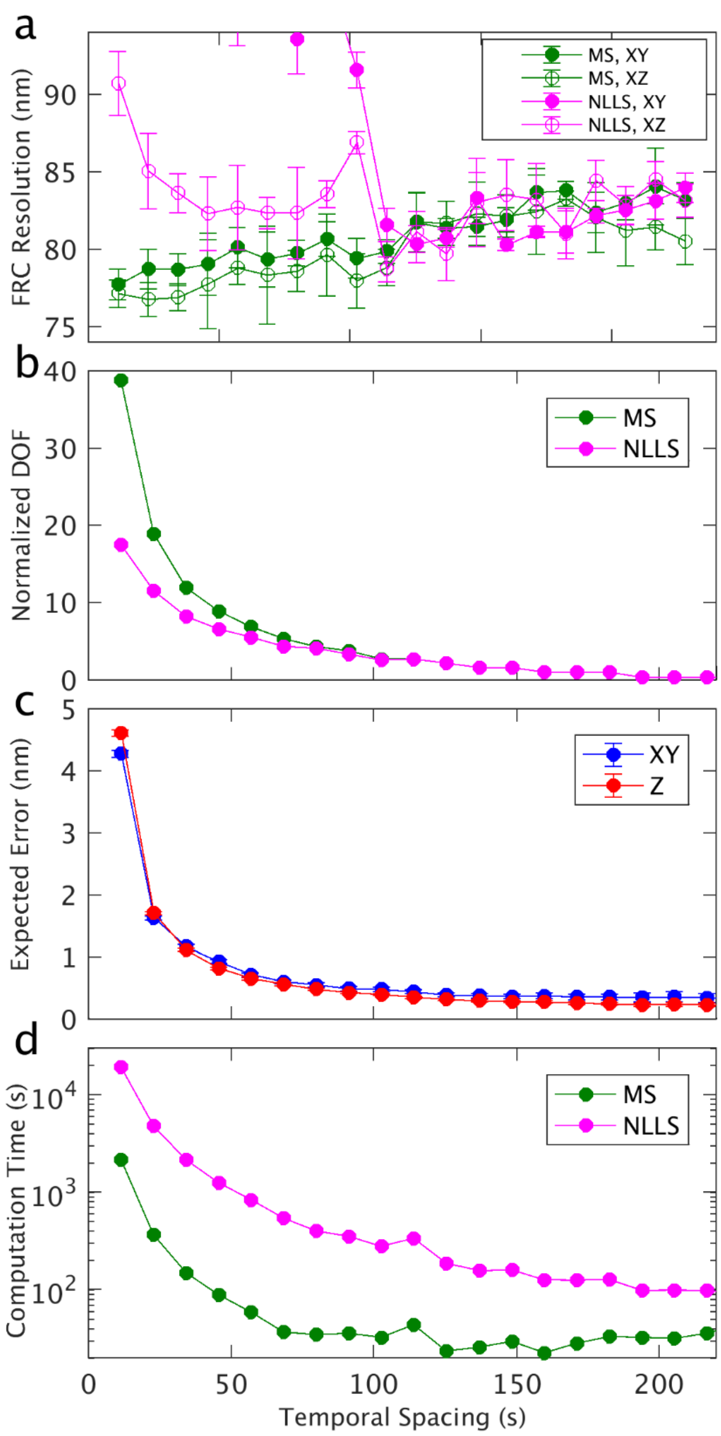

Figure S6. **Drift correction diagnostics for the 3D B cell dataset of Figure 4. a)** FRC resolutions. Error bars are given by the standard deviation over five trials. **b)** The number of degrees of freedom (DOF) after removing outliers (normalized by the number of parameters) for the least square minimization calculation. **c)** RMSE of the lateral (x-y) and axial (z) expected errors for the mean shift calculation of pairwise shifts. Error bars are given by the standard error of the mean. **d)** Total computation time.

Supplemental References

1. Baddeley, A., E. Rubak, and R. Turner. 2016. Spatial point patterns: methodology and applications with R. Boca Raton ; London ; New York: CRC Press, Taylor & Francis Group.

2. Fukunaga, K., and L. Hostetler. 1975. The estimation of the gradient of a density function, with applications in pattern recognition. *IEEE Trans. Inform. Theory*. 21:32–40.

3. Yizong Cheng. 1995. Mean shift, mode seeking, and clustering. *IEEE Transactions on Pattern Analysis and Machine Intelligence*. 17:790–799.

4. Comaniciu, D., and P. Meer. 2002. Mean shift: a robust approach toward feature space analysis. *IEEE Transactions on Pattern Analysis and Machine Intelligence*. 24:603–619.

5. Wang, Y., J. Schnitzbauer, Z. Hu, X. Li, Y. Cheng, Z.-L. Huang, and B. Huang. 2014. Localization events-based sample drift correction for localization microscopy with redundant cross-correlation algorithm. *Opt. Express*. 22:15982.

6. Nieuwenhuizen, R.P.J., K.A. Lidke, M. Bates, D.L. Puig, D. Grünwald, S. Stallinga, and B. Rieger. 2013. Measuring image resolution in optical nanoscopy. *Nat Methods*. 10:557–562.

7. Banterle, N., K.H. Bui, E.A. Lemke, and M. Beck. 2013. Fourier ring correlation as a resolution criterion for super-resolution microscopy. *Journal of Structural Biology*. 183:363–367.

8. Hilgenberg, L.G.W., and M.A. Smith. 2007. Preparation of Dissociated Mouse Cortical Neuron Cultures. *J Vis Exp*.

9. Pappas, S.S., C.-C. Liang, S. Kim, C.O. Rivera, and W.T. Dauer. 2018. TorsinA dysfunction causes persistent neuronal nuclear pore defects. *Human Molecular Genetics*. 27:407–420.

10. Haughton, G., L.W. Arnold, G.A. Bishop, and T.J. Mercolino. 1986. The CH Series of Murine B Cell Lymphomas: Neoplastic Analogues of Ly-1+ Normal B Cells. *Immunological Reviews*. 93:35–52.

11. Stone, M.B., S.A. Shelby, M.F. Núñez, K. Wisser, and S.L. Veatch. 2017. Protein sorting by lipid phase-like domains supports emergent signaling function in B lymphocyte plasma membranes. *eLife Sciences*. 6:e19891.

12. Izeddin, I., J. Boulanger, V. Racine, C.G. Specht, A. Kechkar, D. Nair, A. Triller, D. Choquet, M. Dahan, and J.B. Sibarita. 2012. Wavelet analysis for single molecule localization microscopy. *Opt. Express, OE*. 20:2081–2095.

13. Smith, C.S., N. Joseph, B. Rieger, and K.A. Lidke. 2010. Fast, single-molecule localization that achieves theoretically minimum uncertainty. *Nature Methods*. 7:373–375.

14. Li, Y., M. Mund, P. Hoess, J. Deschamps, U. Matti, B. Nijmeijer, V.J. Sabinina, J. Ellenberg, I. Schoen, and J. Ries. 2018. Real-time 3D single-molecule localization using experimental point spread functions. *Nature Methods*. 15:367–369.
