# Supplementary material for: A mean shift algorithm for drift correction in localization microscopy
Source: Biophys Rep (N Y). 2021 Jul 24;1(1):100008. doi: 10.1016/j.bpr.2021.100008 (PMC8978553; doi:10.1016/j.bpr.2021.100008)
Supplement: Document S2. Article plus supporting material [file mmc2.pdf]

Biophysical Reports

REPORT

A mean shift algorithm for drift correction in localization microscopy

Frank J. Fazekas,[1] Thomas R. Shaw,[1,2] Sumin Kim,[3] Ryan A. Bogucki,[1] and Sarah L. Veatch[1,2,*]

[1]Program in Biophysics, [2]Program in Applied Physics, and [3]Cellular and Molecular Biology Graduate Program, University of Michigan, Ann Arbor, Michigan

ABSTRACT Single-molecule localization microscopy techniques transcend the diffraction limit of visible light by localizing isolated emitters sampled stochastically. This time-lapse imaging necessitates long acquisition times, over which sample drift can become large relative to the localization precision. Here, we present an efficient and robust method for estimating drift, using a simple peak-finding algorithm based on mean shifts that is effective for single-molecule localization microscopy in two or three dimensions.

WHY IT MATTERS Drift correction is a regular part of the analysis pipeline in super-resolution fluorescence localization microscopy because the microscope stage typically moves farther than the localization precision over the time needed to acquire an image. This work presents a mathematically simple mean shift (MS) algorithm that allows for accurate drift correction with high time resolution from acquired localizations in two or three dimensions.

INTRODUCTION

Stochastic super-resolution microscopy techniques, such as STORM (1,2) and PALM (3,4), exploit photoswitching of fluorescent probes to enable imaging of densely labeled samples with resolutions an order of magnitude smaller than the diffraction limit of visible light. Sparsely distributed point spread functions (PSFs) of single emitters are identified in individual image frames, and their centroids are determined according to an appropriate fitting algorithm. The axial position of molecules can be encoded in their PSFs through engineering measures utilizing astigmatism (5,6), multifocal plane imaging (7), or a double helix PSF (8). The final reconstruction is typically a two-dimensional (2D) or three-dimensional (3D) histogram of these single-molecule positions.

Drift due to thermal expansion or mechanical instabilities can degrade image quality over the course of image acquisition, which typically occurs on the timescale of minutes. Drift compensation requires either active stabilization of the microscope (9–13) or a posteriori computation of the drift curves, either using fiducial markers (14–18) or the acquired single-molecule localizations (19–26). In this report, we present a mathematically simple approach to drift correction using a mean shift (MS) algorithm (27–29) for static single-molecule localization microscopy (SMLM) data sets without fiducial markers, with some advantages over past approaches that use nonlinear least-squares (NLLS) fitting of image-based cross-correlations (19–21).

RESULTS

A graphical illustration of the MS algorithm as applied to sample 2D localizations is presented in Fig. 1. The localizations all lie in one of two data sets which sample the same uniformly distributed emitters, but with a constant relative shift $\mathbf{r}_{shift}$ in space. The first step of the algorithm is to extract pairwise displacements between all localizations across the two data sets. When individual displacements are plotted as points (Fig. 1 *b*), displacements arising from the same labeled objects (magenta points) cluster around $\mathbf{r}_{shift}$, whereas displacements arising from different objects (green points) distribute randomly over space. The MS algorithm determines the center of the peak of the distribution through iteration (27–29). At each iteration, all pairs within the radius of consideration are extracted,

Submitted May 13, 2021, and accepted for publication July 20, 2021.

*Correspondence: sveatch@umich.edu

Frank J. Fazekas and Thomas R. Shaw contributed equally to this work.

Editor: Ulrike Endesfelder.

https://doi.org/10.1016/j.bpr.2021.100008

© 2021 The Author(s).

This is an open access article under the CC BY license (http://creativecommons.org/licenses/by/4.0/).

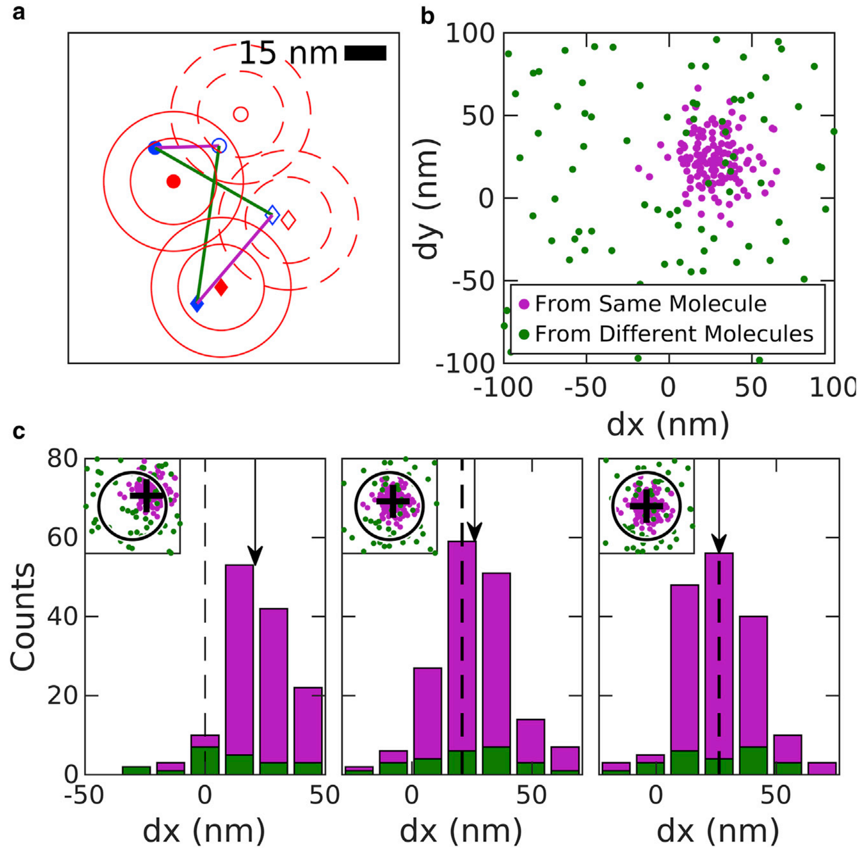

FIGURE 1 Demonstration of the MS algorithm. (*a*) A sample 2D image containing two molecules (filled *red symbols*) that are each localized one time (filled *blue symbols*). At a later time, the molecules are translated 25 nm in both dimensions (*open red symbols*) and two additional localizations are acquired (*open blue symbols*). Red contours indicate one and two times the localization precision around molecule centers. Straight lines show displacements between localizations acquired at distinct times. Some connect localizations from the same displaced molecule (*magenta*), whereas others connect different molecules (*green*). (*b*) Displacements like those shown in (*a*) displayed as points. Points connecting the same molecules cluster around the displacement, whereas points connecting different molecules produce a uniform background. (*c*) Three iterations of the MS algorithm showing the displacements as histograms in one dimension and as points in 2D in the inset. (*Left*) Initially, a region of interest (*circle in inset*) is centered at zero shift. The mean displacement of this subset of points is found (*arrow in main graph* and *cross in inset*). (*Middle*) A new region of interest is drawn around the mean from the initial iteration. The mean displacement from this subset of points (*arrow*) is shifted to slightly more positive values than the previous mean. (*Right*) At the final iteration, the tabulated mean (*arrow*) is equivalent to the starting point (*dashed line*) because the peak is centered within the region of interest.

and the updated shift estimate is the centroid of these pairs. The uniformly distributed background will tend to bias the centroid toward the center of the observation window, whereas the peak moves the mean toward $\mathbf{r}_{shift}$. The observation window is then redrawn around the new mean and the process is repeated until the peak is centered in the observation window. Three iterations of the algorithm are visualized in Fig. 1 *c*.

Although the emitters of Fig. 1 are distributed uniformly in space, leading to the uniform distribution of the pairs from different emitters, the MS method does not depend on this assumption. In samples in which emitters are organized into structures or randomly clustered, the pairs arising from different emitters are also more likely to be at shorter distances, so that the distribution of green points in Fig. 1 *b* will also be peaked at $\mathbf{r}_{shift}$. However, in our experience, pairs of localizations from the same emitter are more important for the MS and other drift estimates. We also note that our analysis assumes that emitters that are localized in one data set remain within the field of view in the second data set, and vice versa. This may not always be the case, and could in principle lead to bias in shift estimates, but in practice this is typically a negligible effect. Roughly, the contribution of fluorophores near the edge of the field of view may be biased by up to about the localization precision, and the fraction of fluorophores that are affected is restricted to those that lie within about a localization precision from the edge of the field of view, in the direction of the drift. So, for example, in a 100 μm field of view with localization precision of 15 nm, we would expect this bias to be on the order of a picometer.

To benchmark this MS approach, we evaluated the ability of the algorithm to detect known shifts of simulated data sets of a circular test cell, as summarized in Fig. 2. Shifts were estimated by both the MS algorithm and by NLLS fitting of a Gaussian to the spatial cross-correlation function of the two data sets, as implemented in the supporting software provided with (21). The performance of each algorithm was similar for easy cases that produce a well-defined peak at $\mathbf{r}_{shift}$. An extremely easy case is depicted at the top of Fig. 2 *a*, in which single molecules are well spaced (surface density $= 5/\mu m^2$) and their positions are well sampled in both frames (twice per molecule on average). In this case, the shift can be clearly identified by eye, and both algorithms

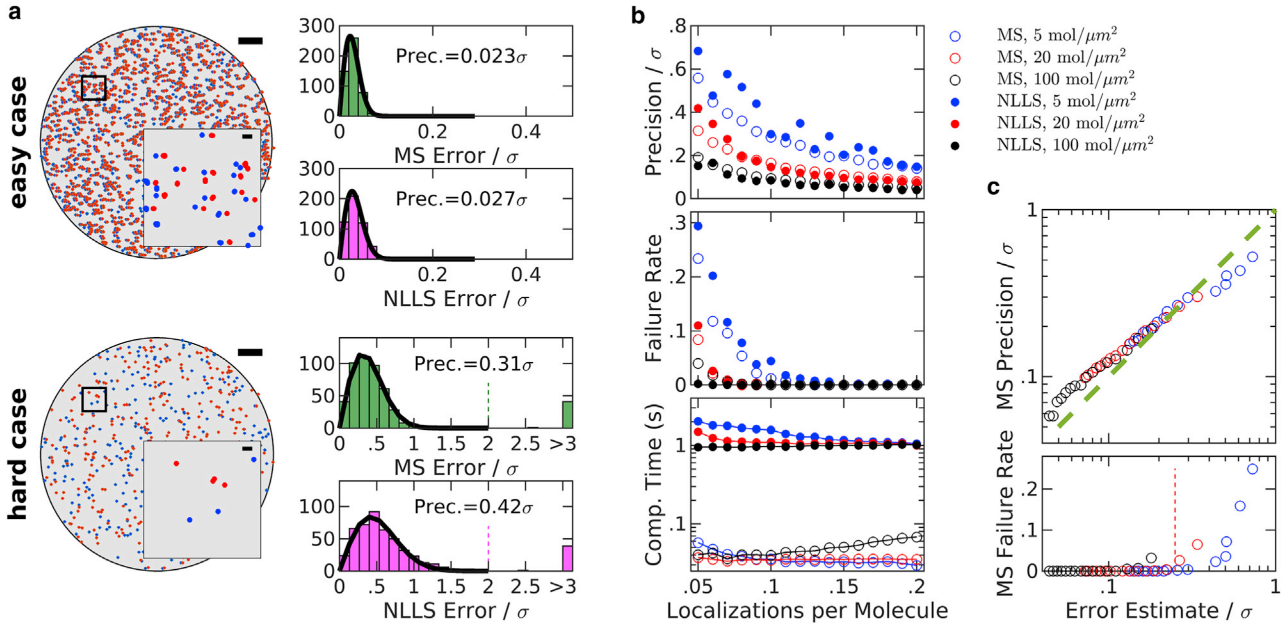

FIGURE 2 Evaluating the MS algorithm on simulated data translated in two dimensions, compared with nonlinear least-squares (NLLS) fitting approach. Simulations and displacement determination approaches are described in Supporting methods and materials. (*a*) Two examples of simulated data sets. Scatter plots on the left show a representative configuration for an extremely easy case, 5 mol/μm^2, with an average of two localizations per molecule (*top*) and a relatively hard case 20 mol/μm^2, with an average of 0.05 localizations per molecule (*bottom*). Scale bars, 2 μm (*large image*) and 150 nm (*inset*). The simulated localization precision is $\sigma = 15$nm. Histograms on the right show errors evaluated for the MS and NLLS approaches for the two cases considered, evaluated from 500 simulations with random displacements between 0 and 150 nm (10σ). The precision of each method (Prec.) is evaluated as the standard deviation (SD) of a Gaussian fit to the central peak of the histogram (*solid line*). The "failure rate" is the fraction of simulations for which the error exceeds twice the localization precision σ, indicated as a dashed line for the hard case. (*b*) Performance comparison of the MS and NLLS approaches over a broad range of simulated conditions. Each point summarizes 500 simulations of the indicated average density and localizations per molecule, with precision and failure rate evaluated as described in (*a*), along with the average computation time per calculation. (*c*) The measured precision plotted as a function of an error in the displacement estimated from data, as derived in Supporting methods and materials. MS failure rate versus error estimate shows that the MS algorithm loses robustness when the estimated error exceeds σ/4 (*dashed line*).

reliably and accurately identify the displacement between frames. The simulation depicted at the bottom of Fig. 2 *a* represents a much harder case, in which molecules are present at higher surface density (20/μm^2) and only 1 in 20 molecules are imaged on average in a given data set. In this case, MS modestly outperforms NLLS fitting, both by locating the peak with improved precision and by more reliably finding the peak overall. These trends hold over simulations conducted over a broad range of molecular densities and localizations per molecule (Fig. 2 *b*). We also estimated shifts from the overall center of mass of each data set, which yielded precisions more than an order of magnitude worse than both the MS and NLLS methods. Moreover, MS is more computationally efficient than NLLS, largely because fast Fourier transforms are not computed in the MS approach. This improvement in speed is enabled through the use of a particularly efficient algorithm from the R package spatstat (30) to extract pairwise displacements between nearby points (see Supporting methods and materials).

For large displacements, both the MS and NLLS algorithms applied in Fig. 2 require an initial step to identify an approximate starting point for the higher accuracy calculation. Fig. S1 shows the failure rate of each algorithm as a function of the distance of the start point from the true shift. MS robustly identifies the main peak over a broad range of simulation conditions as long as it resides within the initial observation window, so large shifts can be identified simply using a large window in the first iteration. This window is typically 100 nm for experimental localizations and 150 nm for the simulations of Fig. 2. NLLS robustly identifies the main peak when the starting point for the computation falls within the localization precision of the peak of the cross-correlation function. In many practical cases, the peak is much farther from the origin than the localization precision, so a separate method is needed to identify a suitable starting point. Here, this is accomplished using a particularly effective algorithm that identifies the global maximum in a smoothed cross-correlation function, as described in the supporting material of (21). The robustness of the NLLS fitting approach is

dependent on the ability of this algorithm to identify a suitable starting point over a broad range of simulation conditions. Note that data sets for which the emitter distribution is highly structured or clustered typically lead to improved performance of the start point identification routine by introducing a broad peak in the cross-correlation function in addition to the sharp peak that represents repeat localizations of the same fluorophore.

This MS approach is applied to SMLM localizations that experience continuous drift by distributing localizations into nonoverlapping temporal bins with equal numbers of frames, and displacement estimates are tabulated between all possible pairs of bins. The number of frames in each temporal bin is an important parameter; short temporal bins have few localizations per molecule, so individual displacements may be estimated imprecisely. Long temporal bins have more localizations per molecule and more precise drift estimates but reduce the time resolution of the drift estimate. A linear least-squares fitting algorithm is then used to generate a trajectory that passes through control points positioned, at times, centered on each temporal bin, as described previously (21), taking advantage of the high redundancy to improve precision of the control points. We have slightly modified this past approach by including weights in the linear least-squares fitting, where weights are determined directly from data using a relation that approximates error in the mean displacement (described in Supporting methods and materials), as demonstrated in simulated data sets (Fig. 2 *c*). Briefly, errors are reduced when there are more pairs originating from the same molecules (*magenta points* in Fig. 1) and errors increase when more pairs originate from different molecules within the observation window (*green points* in Fig. 1). Estimated errors can also act as a proxy for overall reliability of the algorithm. Fig. 2 *c* also shows that MS reliably finds the desired peak when the estimated error remains smaller than one-quarter of the localization precision. This observation can act as a guide when selecting the number of frames included in temporal bins.

It is tempting to distribute frames into overlapping temporal bins, which in principle could improve time resolution while retaining a sufficient number of localizations to accurately determine displacements. However, we find that drift estimates from overlapping time bins are subject to substantial bias, underestimating the actual displacements accrued over time (Fig. S2 *a*). This occurs because the same localizations are present in adjacent bins, biasing the result toward $\mathbf{r}_{\text{shift}} = 0$. Similar bias can arise even in the absence of overlapping time bins because SMLM data frequently contain time-correlated localizations arising from the finite off rates of fluorescent blinking (PALM/dSTORM) or binding (PAINT). These factors mean that pairs of localizations from the same fluorophore are mostly from time separations that are shorter than the time difference between the bin centers and therefore underestimate the average drift between the bins.

The MS approach is applied to a 2D experimental data set of nuclear pore complexes (NPCs) in Fig. 3. NPC assemblies are labeled with primary and secondary antibodies against Nup210 within the nuclear envelope of intact primary mouse neurons, and Fig. 3, *a–d* shows reconstructed images at various magnifications. Fig. 3 *b* is a reconstruction produced without drift correction, in which localizations from single NPCs are smeared over a large area, highlighting the importance of drift correction.

The performance of the MS algorithm was tested on this data set by generating multiple drift trajectories through binning with different temporal resolutions. These trajectories were each applied to the full SMLM data set, and Fourier ring correlation (FRC) (31,32) was used to quantify image resolution (Fig. 3 *e*). For comparison, we conducted drift corrections using the redundant cross-correlation NLLS approach, as described previously (21). In this case, MS modestly outperforms NLLS fitting, allowing for accurate drift correction with smaller temporal bins and modestly improving the resolution of the reconstructed image. We used this data set to explore possible bias introduced because of temporal correlations of single-fluorophore blinking by running the linear least-squares algorithm and including or excluding adjacent pairs of bins on the data of Fig. 2. We found no significant difference between the two cases (Fig. S2 *b*), indicating that the impact of this bias is negligible within experimental errors. Additional diagnostics for the MS and NLLS approaches are shown in Fig. S3.

Drift trajectories are shown in Fig. 3 *f* for temporal bin widths that produce accurate FRC metrics for the MS and NLLS approaches. For MS, a temporal bin slightly larger than the minimum from the FRC curve is used because this produces smaller errors on individual control points. As expected, the drift trajectories follow the same general shape, but the trajectory generated from MS has improved time resolution. In parts of the trajectory, the errors of the control points are smaller than the distance between the trajectories. In these regions, higher time resolution yields improved spatial resolution in the final reconstructed image. Although the differences in the trajectories are significant, their impact is not apparent when viewing reconstructed images of entire nuclei or collections of NPCs, as in Fig. 3, *a* and *c*. Differences become more apparent in images of

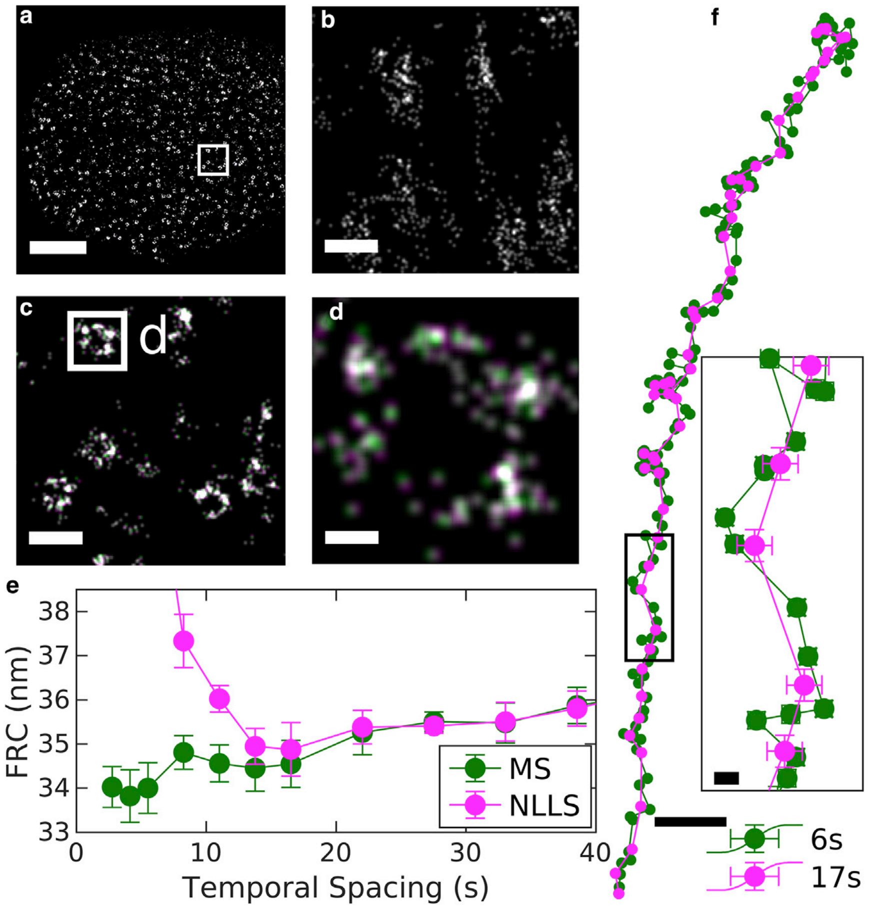

FIGURE 3 Demonstration of MS drift correction of a 2D SMLM data set of antibody-labeled Nup210 in nuclear pore complexes (NPC) within the nuclear envelope of primary mouse neurons. This data set contained 15,500 image frames acquired over 14 min, with an average localization precision of 8 nm. (*a*) Reconstructed image of a single nucleus that is a subset of this data set. (*b*) Reconstruction without drift correction of the region shown within the white box in (*a*). (*c*) Same region as in (*b*) but with drift correction. (*d*) An image of a single complex demonstrates the modest shifts in localizations due to drift corrections estimated with MS and 6 s (*green*) or NLLS and 17 s (*magenta*) temporal bins. Scale bars, 2 μm (*a*), 200 nm (*b* and *c*), and 30 nm (*d*). (*e*) Fourier ring correlation (FRC) resolution after applying drift corrections estimated using the specified temporal bin widths for the MS and NLLS methods. Error bars represent the SD over 20 replicates of the FRC calculation. (*f*) Estimated drift trajectories evaluated from using the method and temporal spacing specified. Error bars represent 68% confidence intervals from the weighted least-squares drift estimation of each control point. Scale bars, 20 nm for the overall drift curve and 2 nm in the inset.

individual pores, in which displacements of several nanometers shift the relative positions of labeled subunits (Fig. 3 *d*).

The MS algorithm is easily extended to localizations acquired in 3D, in which performance improvements are more evident compared with the established NLLS approach. Because the MS algorithm uses points instead of reconstructed images and fast Fourier transforms, it can be extended into 3D without needing expanded memory resources that limit the practical application of NLLS in 3D. Instead, the 3D application of NLLS drift correction is typically accomplished by generating 2D projections that contain less information than the 3D localizations from which they are produced (21). To see why, consider a pair of emitters that are close together in *x-y* but far apart in *z*. Pairs of localizations from this pair of fluorophores will be included in 2D MS drift estimation when using data projected into the *x-y* plane but excluded from the full 3D drift estimation method by virtue of their large separation in *z*. We compare the precision and robustness of 3D MS and NLLS on simulated localizations spread over a cylindrical volume in Fig. S4, in which the NLLS correction is performed on projections into the *x-y*, *x-z*, and *y-z* planes, as described in (21). We also directly compare the *x-y* performance of the full 3D MS method with the 2D MS method performed on data projected into the *x-y* plane (Fig. S5).

Fig. 4 applies the MS approach to an experimental SMLM data set of labeled B cell receptors on the ventral membrane of B cells imaged using a phase mask in the emission path to localize fluorophores in 3D (8). As was the case for simulated data sets, the differences in the performance of the MS and NLLS fitting methods are more pronounced than in the 2D data set of Fig. 3. Additional diagnostics for the 3D case are shown in Fig. S6.

In summary, a mathematically simple MS algorithm modestly outperforms cross-correlation-based estimates of drift correction in 2D and more significantly improves the time resolution of drift corrections in 3D. The approach is computationally efficient, is robust without sophisticated methods to estimate starting points, and does not require image reconstruction with memory and pixelation limitations. The metric provided to estimate error and predict robustness directly from data provides users with a means to evaluate the quality of a drift correction within an SMLM analysis pipeline. For the example data sets explored, modest improvements in

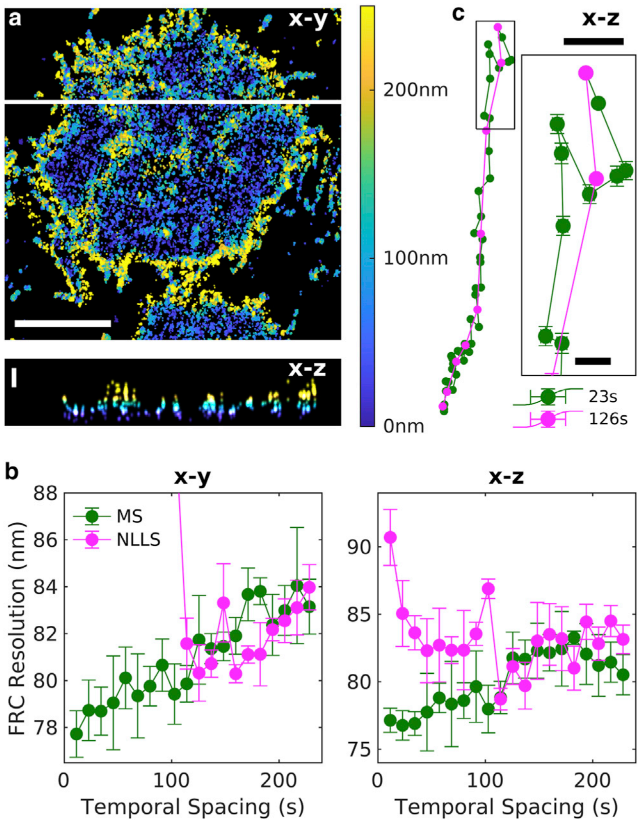

FIGURE 4 Demonstration of MS drift correction of a 3D SMLM data set of B cell receptors at the ventral plasma membrane of CH27 B cells. This data set contained more than 400,000 localizations acquired over 15 min, with an average localization precision of 17 nm in the lateral (*x-y*) dimension and 31 nm in the axial (*z*) dimension. (*a*) Reconstructed image of a subset of this data set showing the average z position within each *x-y* pixel, as indicated in the color bar. *x-z* slice at the position drawn as a white line is shown below. Scale bars, 5 μm for *x-y* and 200 nm for *z*. (*b*) Fourier ring correlation (FRC) estimates of image resolution after applying drift corrections estimated using the specified temporal bin widths for the MS and NLLS methods. Error bars represent the SD over five replicates of the FRC calculation. (*c*) Estimated drift trajectories evaluated with the specified temporal spacing. Error bars represent 68% confidence intervals from the weighted least-squares drift estimation of each control point. Scale bars, 50 nm (10 nm in the inset).

resolution lead to adjustments of localized molecule positions relevant for evaluating the structure of protein complexes in cells.

SUPPORTING MATERIAL

Supporting material can be found online at https://doi.org/10.1016/j.bpr.2021.100008.

AUTHOR CONTRIBUTIONS

The method was devised by T.R.S. in consultation with F.J.F. and S.L.V. F.J.F. wrote the majority of the code and performed the majority of analyses in consultation with T.R.S. and S.L.V. F.J.F., T.R.S., and S.L.V. wrote the text. S.K. prepared and imaged nuclear pore complex samples for Fig. 3. R.A.B. prepared and imaged B cell samples for Fig. 4.

DECLARATION OF INTERESTS

The authors declare no competing interests.

ACKNOWLEDGMENTS

We thank Kathleen Wisser and Sarah Shelby for assistance with experiments.

This work was supported by grants from the U.S. National Science Foundation (MCB1552439) and National Institutes of Health (GM129347 and GM110052).

SUPPORTING CITATIONS

References (33–39) appear in the Supporting material.

REFERENCES

1. Rust, M. J., M. Bates, and X. Zhuang. 2006. Sub-diffraction-limit imaging by stochastic optical reconstruction microscopy (STORM). *Nat. Methods.* 3:793–795.
2. Heilemann, M., S. van de Linde, ..., M. Sauer. 2008. Subdiffraction-resolution fluorescence imaging with conventional fluorescent probes. *Angew. Chem. Int. Ed. Engl.* 47:6172–6176.
3. Betzig, E., G. H. Patterson, ..., H. F. Hess. 2006. Imaging intracellular fluorescent proteins at nanometer resolution. *Science.* 313:1642–1645.
4. Hess, S. T., T. P. K. Girirajan, and M. D. Mason. 2006. Ultra-high resolution imaging by fluorescence photoactivation localization microscopy. *Biophys. J.* 91:4258–4272.
5. Kao, H. P., and A. S. Verkman. 1994. Tracking of single fluorescent particles in three dimensions: use of cylindrical optics to encode particle position. *Biophys. J.* 67:1291–1300.
6. Holtzer, L., T. Meckel, and T. Schmidt. 2007. Nanometric three-dimensional tracking of individual quantum dots in cells. *Appl. Phys. Lett.* 90:053902.
7. Juette, M. F., T. J. Gould, ..., J. Bewersdorf. 2008. Three-dimensional sub-100 nm resolution fluorescence microscopy of thick samples. *Nat. Methods.* 5:527–529.
8. Pavani, S. R. P., M. A. Thompson, ..., W. E. Moerner. 2009. Three-dimensional, single-molecule fluorescence imaging beyond the diffraction limit by using a double-helix point spread function. *Proc. Natl. Acad. Sci. USA.* 106:2995–2999.
9. Carter, A. R., G. M. King, ..., T. T. Perkins. 2007. Stabilization of an optical microscope to 0.1 nm in three dimensions. *Appl. Opt.* 46:421–427.
10. Grover, G., W. Mohrman, and R. Piestun. 2015. Real-time adaptive drift correction for super-resolution localization microscopy. *Opt. Express.* 23:23887–23898.
11. Tafteh, R., L. Abraham, ..., K. C. Chou. 2016. Real-time 3D stabilization of a super-resolution microscope using an electrically tunable lens. *Opt. Express.* 24:22959–22970.
12. Schmidt, P. D., B. H. Reichert, ..., S. Sivasankar. 2018. Method for high frequency tracking and sub-nm sample stabilization in single molecule fluorescence microscopy. *Sci. Rep.* 8:13912.
13. Coelho, S., J. Baek, ..., K. Gaus. 2021. 3D active stabilization for single-molecule imaging. *Nat. Protoc.* 16:497–515.

14. Huang, B., W. Wang, …, X. Zhuang. 2008. Three-dimensional super-resolution imaging by stochastic optical reconstruction microscopy. *Science*. 319:810–813.

15. Bon, P., N. Bourg, …, S. Lévêque-Fort. 2015. Three-dimensional nanometre localization of nanoparticles to enhance super-resolution microscopy. *Nat. Commun.* 6:7764.

16. Colomb, W., J. Czerski, …, S. K. Sarkar. 2017. Estimation of microscope drift using fluorescent nanodiamonds as fiducial markers. *J. Microsc.* 266:298–306.

17. Ma, H., J. Xu, …, Y. Liu. 2017. A simple marker-assisted 3D nanometer drift correction method for superresolution microscopy. *Biophys. J.* 112:2196–2208.

18. Balinovic, A., D. Albrecht, and U. Endesfelder. 2019. Spectrally red-shifted fluorescent fiducial markers for optimal drift correction in localization microscopy. *J. Phys. D Appl. Phys.* 52:204002.

19. Mlodzianoski, M. J., J. M. Schreiner, …, J. Bewersdorf. 2011. Sample drift correction in 3D fluorescence photoactivation localization microscopy. *Opt. Express*. 19:15009–15019.

20. Geisler, C., T. Hotz, …, A. Egner. 2012. Drift estimation for single marker switching based imaging schemes. *Opt. Express*. 20:7274–7289.

21. Wang, Y., J. Schnitzbauer, …, B. Huang. 2014. Localization events-based sample drift correction for localization microscopy with redundant cross-correlation algorithm. *Opt. Express*. 22:15982–15991.

22. Elmokadem, A., and J. Yu. 2015. Optimal drift correction for superresolution localization microscopy with Bayesian inference. *Biophys. J.* 109:1772–1780.

23. Schlangen, I., J. Franco, …, C. Rickman. 2016. Marker-less stage drift correction in super-resolution microscopy using the single-cluster PHD filter. *IEEE J. Sel. Top. Signal Process.* 10:193–202.

24. Han, R., L. Wang, …, F. Zhang. 2015. Drift correction for single-molecule imaging by molecular constraint field, a distance minimum metric. *BMC Biophys.* 8:1.

25. Wester, M. J., D. J. Schodt, …, K. A. Lidke. 2021. Robust, fiducial-free drift correction for super-resolution imaging. *bioRxiv* https://doi.org/10.1101/2021.03.26.437196.

26. Cnossen, J., T. J. Cui, …, C. Smith. 2021. Drift correction in localization microscopy using entropy minimization. *Opt. Express*.

27. Fukunaga, K., and L. Hostetler. 1975. The estimation of the gradient of a density function, with applications in pattern recognition. *IEEE Trans. Inf. Theory*. 21:32–40.

28. Cheng, Y. 1995. Mean shift, mode seeking, and clustering. *IEEE Trans. Pattern Anal. Mach. Intell.* 17:790–799.

29. Comaniciu, D., and P. Meer. 2002. Mean shift: a robust approach toward feature space analysis. *IEEE Trans. Pattern Anal. Mach. Intell.* 24:603–619.

30. Baddeley, A., E. Rubak, and R. Turner. 2016. Spatial Point Patterns: Methodology and Applications with R. CRC Press, Taylor & Francis Group, Boca Raton; London; New York.

31. Nieuwenhuizen, R. P. J., K. A. Lidke, …, B. Rieger. 2013. Measuring image resolution in optical nanoscopy. *Nat. Methods*. 10:557–562.

32. Banterle, N., K. H. Bui, …, M. Beck. 2013. Fourier ring correlation as a resolution criterion for super-resolution microscopy. *J. Struct. Biol.* 183:363–367.

33. Hilgenberg, L. G. W., and M. A. Smith. 2007. Preparation of dissociated mouse cortical neuron cultures. *J. Vis. Exp.* 2007:562.

34. Pappas, S. S., C.-C. Liang, …, W. T. Dauer. 2018. TorsinA dysfunction causes persistent neuronal nuclear pore defects. *Hum. Mol. Genet.* 27:407–420.

35. Haughton, G., L. W. Arnold, …, T. J. Mercolino. 1986. The CH series of murine B cell lymphomas: neoplastic analogues of Ly-1+ normal B cells. *Immunol. Rev.* 93:35–51.

36. Stone, M. B., S. A. Shelby, …, S. L. Veatch. 2017. Protein sorting by lipid phase-like domains supports emergent signaling function in B lymphocyte plasma membranes. *eLife*. 6:e19891.

37. Izeddin, I., J. Boulanger, …, J. B. Sibarita. 2012. Wavelet analysis for single molecule localization microscopy. *Opt. Express*. 20:2081–2095.

38. Smith, C. S., N. Joseph, …, K. A. Lidke. 2010. Fast, single-molecule localization that achieves theoretically minimum uncertainty. *Nat. Methods*. 7:373–375.

39. Li, Y., M. Mund, …, J. Ries. 2018. Real-time 3D single-molecule localization using experimental point spread functions. *Nat. Methods*. 15:367–369.

Biophysical Reports, Volume *1*

Supplemental information

A mean shift algorithm for drift correction in localization microscopy

Frank J. Fazekas, Thomas R. Shaw, Sumin Kim, Ryan A. Bogucki, and Sarah L. Veatch

Supplemental Methods and Materials

Simulated Datasets

An idealized 2D SMLM dataset was simulated as a spatially random set of fluorophores on a 20μm diameter circular cell, with each fluorophore giving rise to a Poisson-distributed number of localizations with isotropic Gaussian localization error $\sigma_x = \sigma_y = 15/\sqrt{2}$ nm. We define $\sigma = \sqrt{\sigma_x^2 + \sigma_y^2} = 15$ to denote the total root-mean-square localization error. A second dataset was generated from the same fluorophore locations, localization precision, and average number of localizations per fluorophore, and shifted between 0 and 150 nm in a random direction. Simulated datasets were generated over a range of densities (5 to 100 per μm^2) and a range of localizations per molecule (.05 to .2).

An idealized 3D SMLM dataset was simulated in a similar fashion. Fluorophores were distributed uniformly on a cylinder 20 μm in diameter and 2 μm deep. Each fluorophore produces a Poisson-distributed number of localizations with $\sigma_x = \sigma_y = 15/\sqrt{2}$ nm as before, and with $\sigma_z = 30/\sqrt{2}$ nm. One dataset is translated by a random distance between 0 and 150 nm in a random direction in x, y, and z.

Extracting Close Pairs of Coordinates Between Datasets

Consider two point sets $\mathbf{u}_i = (u_{ix}, u_{iy})$ and $\mathbf{v}_j = (v_{jx}, v_{jy})$, for $i = 1,...,n_1$ and $j = 1,...,n_2$. We wish to quickly determine which pairs (i, j) are closer than some maximum distance $r_{\max}$; i.e. which pairs satisfy $\|\mathbf{u}_i - \mathbf{v}_j\| < r_{\max}$. The algorithm is adapted from the code for the `closepairs()` and `crosspairs()` functions of the R package spatstat (1), and implemented in C with a MATLAB interface. We first sort each dataset with respect to its x-coordinate, so that $u_{kx} \le u_{lx}$ whenever $k \le l$. Then the algorithm proceeds as follows:

1. Let $i = 1$ and $j_{\text{left}} = 1$.
2. Let $x_{\text{left}} = u_{ix} - r_{\max}$. All close pairs of $\mathbf{u}_i$ must satisfy $v_{jx} > x_{\text{left}}$.
3. Increment j_{left} until $v_{j_{\text{left}}x} \ge x_{\text{left}}$.

4. For each $j = j_{left},\ldots,n_2$, if $v_{jx} - u_{ix} > r_{\max}$, increment i and return to step 2. Otherwise, compute $r_{ij}^2 = (u_{ix} - v_{ix})^2 + (u_{iy} - v_{iy})^2$. If $r_{ij}^2 \le r_{\max}^2$, add (i, j) to the list of results.

This algorithm avoids computing pairwise distances between most pairs in the dataset, and so is much faster and more memory efficient than a brute force approach. It can be readily adapted to higher dimensions by applying the appropriate *n*-dimensional distance metric in step 4. For convenience, our implementation returns the displacements $\Delta\mathbf{r}_{ij} = \mathbf{v}_j - \mathbf{u}_i$, and total distance $r_{ij} = \|\Delta\mathbf{r}_{ij}\|$ for each pair (i, j), instead of the indices themselves.

Determining Shifts between Translated Datasets Using a Mean Shift Algorithm

Given the set of displacements $\Delta\mathbf{r}_{ij} = \mathbf{u}_i - \mathbf{v}_j$ between two point sets $\mathbf{u}_i$ and $\mathbf{v}_j$, a mean shift clustering algorithm (2–4) can be applied to search for the peak of the displacement density function. Briefly, let $\mathbf{r}_{\text{shift},0}$ be an initial guess to initialize the shift estimate, and δ a radius of consideration to use in the optimization procedure. The algorithm proceeds by iteration, by setting

$$\mathbf{r}_{\text{shift},t+1} = \left\langle \mathbf{r}_{ij} \right\rangle_{\left\|\mathbf{r}_{ij} - \mathbf{r}_{\text{shift},t}\right\| \le \delta},$$

where the average is restricted to the subset of displacements $\mathbf{r}_{ij}$ that satisfy the subscript, i.e. that are within a radius δ from the previous shift estimate $\mathbf{r}_{\text{shift},t}$. The algorithm terminates when the distance $\left\|\mathbf{r}_{\text{shift},t+1} - \mathbf{r}_{\text{shift},t}\right\|$ between subsequent shift estimates becomes smaller than machine precision, or when the number of iterations exceeds a user-defined maximum number. δ must be sufficiently large so that the true shift resides within the explored area when centered at the starting-point. In practice, we apply the algorithm twice: first with a large δ to determine the rough shift, and then with a smaller δ, using the first estimate as a starting point, to refine the estimate.

While the above can be applied directly to 3-dimensional data by taking the average over a 3-dimensional ball of radius δ instead of the 2-dimensional disc, we find it is advisable to consider an ellipsoid that is stretched in the z-direction, to account for the larger axial localization errors present in our 3-dimensional simulated and experimental datasets. In the present work, we let the semimajor axis of the ellipsoid be $\sqrt{2}\delta$, in the z direction, and the semiminor axes both δ, so that x-y cross-sections of the regions of consideration are discs.

Estimates of mean shift error

We model the distribution of pairs around the true shift as a Gaussian-distributed peak with standard deviation ς, centered on the true shift $\mathbf{r}_{\text{shift}}$ on a uniformly distributed background. Assuming $\mathbf{r}_{\text{shift},t}$ is sufficiently close to $\mathbf{r}_{\text{shift}}$ that most of the Gaussian peak falls within the region of consideration, the variance ξ^2 of the two components of $\mathbf{r}_{\text{shift},t}$ is given by

$$\xi^2 = \mathrm{Var}\left[r_{\text{shift},t,x}\right] = \mathrm{Var}\left[r_{\text{shift},t,y}\right] = \frac{n_{\text{true}}\varsigma^2 + n_{\text{false}}\delta^2/4}{(n_{\text{true}} + n_{\text{false}})^2},$$

where δ is the radius of consideration for the MS algorithm, and n_{true} and n_{false} are respectively the number of "true pairs" that are drawn from the Gaussian part of the distribution (displacements between different localizations of the same molecules) and the number of "false pairs" that are drawn from the uniform part (displacements between different molecules), that fall within the region of consideration. Furthermore, the expected value after one more step can be derived:

$$\begin{aligned} \mathrm{E}\left[\mathbf{r}_{\text{shift}.t+1} - \mathbf{r}_{\text{shift},t}\right] &= \frac{n_{\text{false}}\mathbf{r}_{\text{shift}.t} + n_{\text{true}}\mathbf{r}_{\text{shift}}}{n_{\text{true}} + n_{\text{false}}} - \mathbf{r}_{\text{shift,t}} \\ &= \frac{n_{\text{true}}}{n_{\text{true}} + n_{\text{false}}}\left(\mathbf{r}_{\text{shift}} - \mathbf{r}_{\text{shift},t}\right). \end{aligned}$$

Suppose t is the final step of the algorithm, i.e. $\mathbf{r}_{\text{shift},t+1} - \mathbf{r}_{\text{shift},t} = 0$. Then by hypothesis, $\mathbf{r}_{\text{shift},t}$ deviates from its expected value by

$$\frac{n_{\text{true}}}{n_{\text{true}} + n_{\text{false}}}\left\|\mathbf{r}_{\text{shift},t} - \mathbf{r}_{\text{shift}}\right\|.$$

This deviation will typically take on values comparable to the standard deviation ξ shown above. Thus, we estimate the error of the MS algorithm by:

$$\text{Predicted Error} = \frac{\sqrt{n_{\text{true}}\varsigma^2 + n_{\text{false}}\delta^2/4}}{n_{\text{true}}}.$$

This predicted error is to be interpreted as an estimate of the standard deviation of the shift estimate in each direction.

In practice, the parameters n_{true}, n_{false}, and ς are not known, so we estimate them from data. Specifically, we construct the isotropic cross-correlation function $c(r)$ from the pair separations $\mathbf{r}_{ij}$, determine the baseline of $c(r)$ from its long-range median value, and use the baseline to infer n_{true} and n_{false}. Finally, we fit $c(r)$ to a Gaussian plus a constant to estimate ς .This error estimate is derived from a heuristic argument and is not exact. However, its performance is adequate in practice. See Figure 2c for a comparison to observed standard deviations of MS shift estimates.

For 3D data, we compute lateral and axial predicted errors separately, by projecting the data from the ellipsoidal region of consideration into the x-y plane or onto the z axis, respectively. n_{true}, n_{false}, and ς are estimated separately for the lateral and axial directions from the two projections.

Evaluating displacements using nonlinear least squares (NLLS) fitting

Displacements $\mathbf{r}_{\text{shift}}$ between pairs of localization datasets were also estimated by NLLS fitting of a Gaussian to the spatial cross-correlation function of the two datasets. NLLS fitting was accomplished using software published as Supplemental material of (5). Images were first reconstructed from simulated localizations with a pixel size of 15nm for simulated localizations, or from acquired data with a pixel size of 8nm for Nup210 or 15nm for B cell receptor experimental localizations. Cross-correlations are tabulated using 2D Fast Fourier Transforms (FFTs) and then fit a 2D Gaussian function to a subset of the cross-correlation centered at the start-point of the NLLS algorithm. The software from (5) finds the start-point using an elegant smoothing step to reduce noise then uses the largest local maximum of the smoothed cross-correlation as the start-point for fitting.

For localizations acquired in 3D, multiple 2D projections were constructed from 3D localizations, then the procedures described for 2D images were applied to determine displacements. First, images projecting on the lateral dimension (x-y plane) were generated and the lateral displacement was determined. To compute the z displacement, both the xz and yz

projections were used, and the final z displacement was the average determined from the two projections.

Correcting continuous drift

Continuous drift was corrected by temporally dividing the data into N bins, each having the same number of frames. For each of the $N(N-1)/2$ pairs (m,n) of temporal bins, the mean shift or NLLS algorithm is applied to estimate the shift $\mathbf{r}_{\text{shift},m\rightarrow n}$ from temporal bin m to n, corresponding to the drift between the bins. Drift at each of the N time points is calculated from the $N\,(N-1)/2$ pairwise shifts using a least-squares minimization algorithm (5); this takes advantage of the overdetermined nature of the drift calculation to improve the precision of the measurement. Outlier shifts, whose residual with respect to the least-squares estimate exceeds a user-defined threshold, can also be discarded as described in (5). These shifts typically correspond to "failures" of the shift estimation method. The final drift curve at each frame is determined by linear interpolation and extrapolation from the N basis points.

Evaluating performance of displacement algorithms

For simulated localizations, errors away from known displacements were tabulated for each simulated configuration. The 2D precision of each method is defined as the standard deviation of a centered, isotropic 2D Gaussian fit to the central peak of the histogram of these values, considering only values that fall within twice the localization precision (2σ) used in the simulation. The fit is applied directly to the absolute errors $\left\|\mathbf{r}_{\text{shift,est.}}-\mathbf{r}_{\text{shift}}\right\|$ with the distribution function $f(r)=\frac{r}{\sigma^2}e^{-r^2/2\sigma^2}$. Similarly, in the 3D case, x- and z-precision are evaluated separately by fitting 1D Gaussian functions to the x- and z-errors, respectively. Values that fall outside of the 2σ window are reported as failures of the algorithm, and contribute to the failure rates reported in figures. Since failures can return values with large errors, they can have an outsized impact on simpler precision metrics, such as the root mean square error (RMSE). Computation time was assessed in MATLAB using the built-in `tic` and `toc` functions. For simulated data, computation time was averaged over 500 simulations for each condition. For experimental data, computation time includes the $N(N-1)/2$ shift estimates and the error estimates for each shift

estimate in the MS case. Normalized residual degrees of freedom (Normalized DOF) of the linear least squares algorithm are calculated by the ratio of shifts that are used for the final least squares minimization step (after removal of outliers) to the number of time points at which drift is estimated (i.e. $N-1$). This serves as a diagnostic for how much redundancy is included in the linear least squares minimization step.

Evaluating the resolution of drift-corrected datasets

Resolutions of the final reconstructed images were compared using Fourier Ring Correlation (6, 7). Specifically, we used code adapted from the supplementary software of (6). To compute the x-y resolution, nearby localizations belonging to adjacent camera frames were grouped together, with the position taken to be the average of the relevant coordinates. The FRC curves were produced by dividing the dataset into blocks of 500 frames and allocating an equal number of blocks randomly to each of the two sets. The pixel size was taken to be 5nm. For the B cell dataset, the Fourier Ring approach was applied to the xy and xz projections in turn, also using a pixel size of 5nm in each case.

Preparation of cellular samples for imaging

Mouse primary neurons were isolated from P0 mouse pups that were decapitated and brains were isolated into ice cold, filtered dissection buffer (6.85 mM sodium chloride, 0.27mM potassium chloride, 0.0085mM sodium phosphate dibasic anhydrous, 0.011mM potassium phosphate monobasic anhydrous, 33.3mM D-glucose, 43.8mM sucrose, 0.277mM HEPES, pH 7.4) as described in (8). After removing the cerebellum and the meninges, cortices were dissected out, placed into a microcentrifuge tube, and cut into small pieces with dissection forceps. Cortices were incubated in 50μL papain (2mg/mL; BrainBits) and 10μL DNase I (1mg/mL; Worthington Biochemical) for 30min at 37 °C. 500μL BrainPhys Neuronal Medium (Stemcell Technologies) and 10μL additional DNase I were added, and cortices were titurated using P1000 and P200 pipet tips. Titurated cortices were centrifuged at 1000rpm for 5min. After discarding the supernatants, the pellets were titurated and centrifuged three more times until the supernatant remained clear and neuronal pellets were visible. Pelleted neurons were resuspended in BrainPhys Neuronal medium with SM1 supplement as previously described (9), then plated onto 35mm #1.5 glass-bottom dishes (MatTek Life Sciences) coated with polyethlenimine

(100 μg/ml; Polysciences). Neurons were incubated in 5% CO_2 at 37 °C, and 1mL of media was replaced every four days.

On day 10 of culture (days *in vitro* 10), neurons were rinsed with sterile Hank's Balanced Salt Solution, then incubated for 1min with pre-warmed 2% PFA (Electron Microscopy Sciences) in Phosphate Buffered Saline (PBS). The neurons were then incubated in 0.4% Triton X-100 (Millipore Sigma) in PBS for 3min, and fixed for 30min with 2% PFA in PBS. Neurons were then washed with PBS five times, incubated in blocking buffer containing 5% Normal Donkey Serum and 5% Normal Goat Serum (Jackson Laboratories) for 30min, then labeled with Nup210 polyclonal antibody diluted in blocking buffer (1:200; Bethyl laboratories A301-795A) overnight in 4 °C. The following day, neurons were washed three times in PBS then stained with Goat-anti-rabbit Alexafluor 647 secondary antibody (1:1000; Thermo Fisher) for an hour, washed three times with PBS, then imaged.

CH27 B cells (10) were cultured, allowed to adhere to 35mm #1.5 glass-bottom dishes (MatTek Life Sciences) overnight, then incubated in Alexa647 conjugated fAb prior to fixation in 4% PFA and 0.1% gluteraldehide (Electron Microscopy Sciences), as described previously (11). The labeled fAb antibody was prepared by conjugating an Alexa647 NHS ester (ThermoFisher) to an unconjugated fAb (Goat Anti-Mouse IgM, μ chain specific; Jackson Immunoresearch) using established protocols (11).

Single molecule imaging and localization

Imaging was performed using an Olympus IX83-XDC inverted microscope. TIRF laser angles where achieved using a 60X UAPO TIRF objective (NA = 1.49), and active Z-drift correction (ZDC) (Olympus America) as described previously. The ZDC was not used for collection of 3D datasets. Alexa 647 was excited using a 647 nm solid state laser (OBIS, 150 mW, Coherent) coupled in free-space through the back aperture of the microscope. Fluorescence emission was detected on an EMCCD camera (Ultra 897, Andor) after passing through a 2x expander. Imaging in 3D was accomplished using a SPINDLE module equipped with a DH-1 phase mask (DoubleHelix LLC).

Single molecule positions were localized in individual image frames using custom software written in Matlab. Peaks were segmented using a standard wavelet algorithm (12) and segmented peaks were then fit on GPUs using previously described algorithms for 2D (13) or 3D localizations (14). After localization, points were culled to remove outliers prior to drift correction. Images were rendered by generating 2D histograms from localizations followed by convolution with a Gaussian for display purposes.

Supplemental Software:

Supplemental Software can be found at https://github.com/VeatchLab/Mean-Shift-Drift-Correction. The software contains Matlab and C code to run mean shift drift corrections on 2D and 3D SMLM data. We also include slightly modified versions of the NLLS and FRC codes published previously (5, 6). Three example scripts are also included:

1. meanshift_example.m: determine the shift between a single pair of sets of localizations sampling the same structure at different times. The example uses data from the 2D nuclear pore complex (NPC) dataset of Fig 3.
2. example_NPC.m: correct 2D drift from the full NPC dataset of Fig 3.
3. example_Bcell.m: correct 3D drift from the full B cell dataset of Fig 4.

Supplemental Figures:

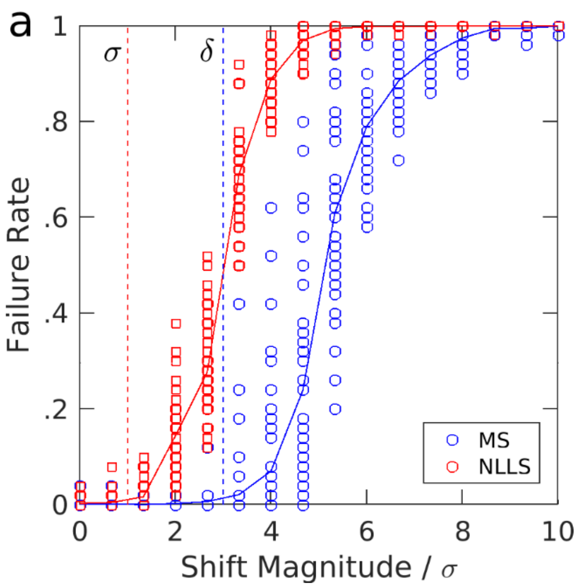

Figure S1. Evaluating the mean shift (MS) and NLLS algorithms with the start-point at the origin. Simulations and shift determination approaches are described in Methods. **a)** Shifts between 0 and 10 times the localization precision (σ) are applied in a random direction, and the MS and NLLS algorithms are applied to determine these displacements. The observation window (δ) for the MS algorithm has an extent of 3σ as indicated by the dashed blue line. The "failure rate" is the fraction of simulations whose error exceeds 2σ. Each point represents a given combination of fluorophore density and number of localizations per molecule, averaged over 50 independent trials. Densities range from 5 to 100 molecules per μm^2, and localizations per molecule range from .05 to .2.

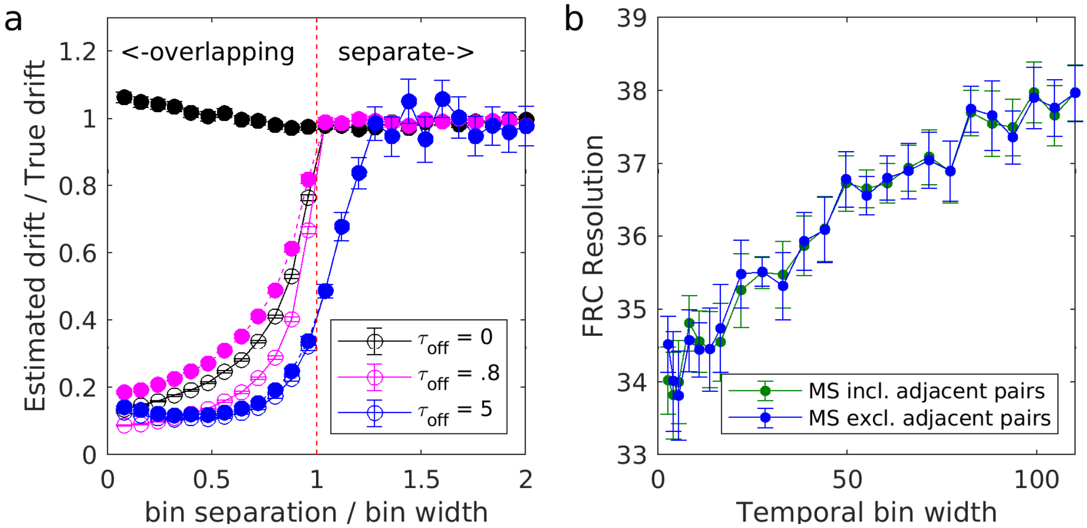

Figure S2. Drift estimates between overlapping temporal bins are prone to bias. a) Mean shift (MS) shift estimates for pairs of bins that are overlapping or separated by short times. Simulations are similar to those for Figure 2 with a cell of radius 10 μm and randomly distributed fluorophores at a density of 20 per μm^2, but with explicit blinking kinetics, modeled as a simple two state (fluorescent/dark) system, with activation and deactivation time constants τ_{on} and τ_{off} for the dark → fluorescent and fluorescent → dark transitions, respectively. A constant drift rate of .1 nm per frame is applied. The drift estimates shown here are for 50 frame temporal bins, with bin starts separated by the bin separation times as shown. For each τ_{off}, τ_{on} is adjusted so that the average number of localizations per fluorophore is approximately 0.15. Open circles represent drift estimates using all pairs of simulated points, including the trivial 0 displacements between points that appear in the overlap of the two temporal bins. Filled circles represent drift estimates using all pairs of distinct point, i.e. excluding the trivial 0 displacements between points that appear in the overlap of the two temporal bins. Note that overlapping bins are subject to substantial bias even for quite short τ_{off} (magenta points), and that even non-overlapping bins may be subject to bias when τ_{off} is long (blue points). b) FRC resolutions for the nuclear pore complex data of Figure 3, with MS drift corrections including or excluding drift estimates for adjacent pairs of temporal bins in the linear least squares fit.

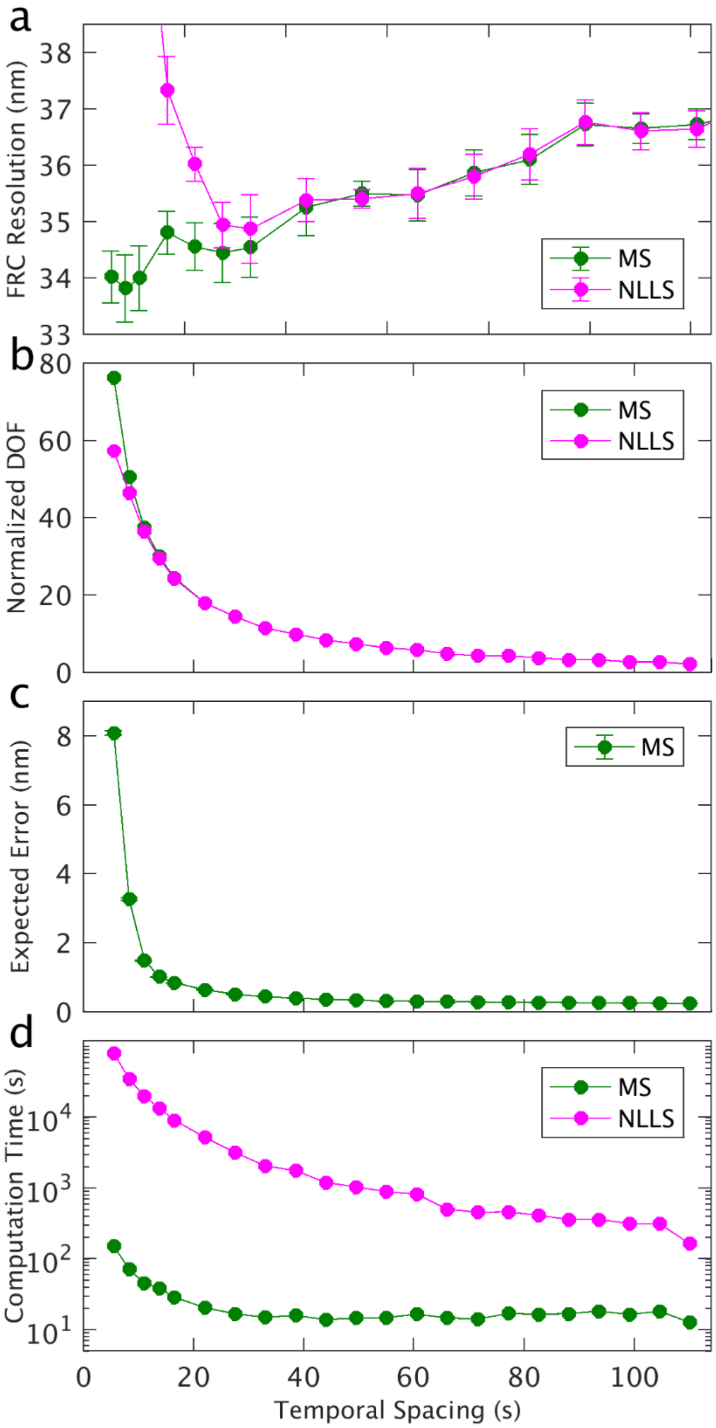

Figure S3. **Drift correction diagnostics for the nuclear pore complex dataset of Figure 3. a)** FRC resolutions. Error bars are given by the standard deviation over 20 trials. **b)** The number of degrees of freedom (DOF) after removing outliers (normalized by the number of parameters) for the redundant least square minimization calculation. **c)** RMSE of the expected errors for the mean shift method. Error bars are given by the standard error of the mean. **d)** Total computation time.

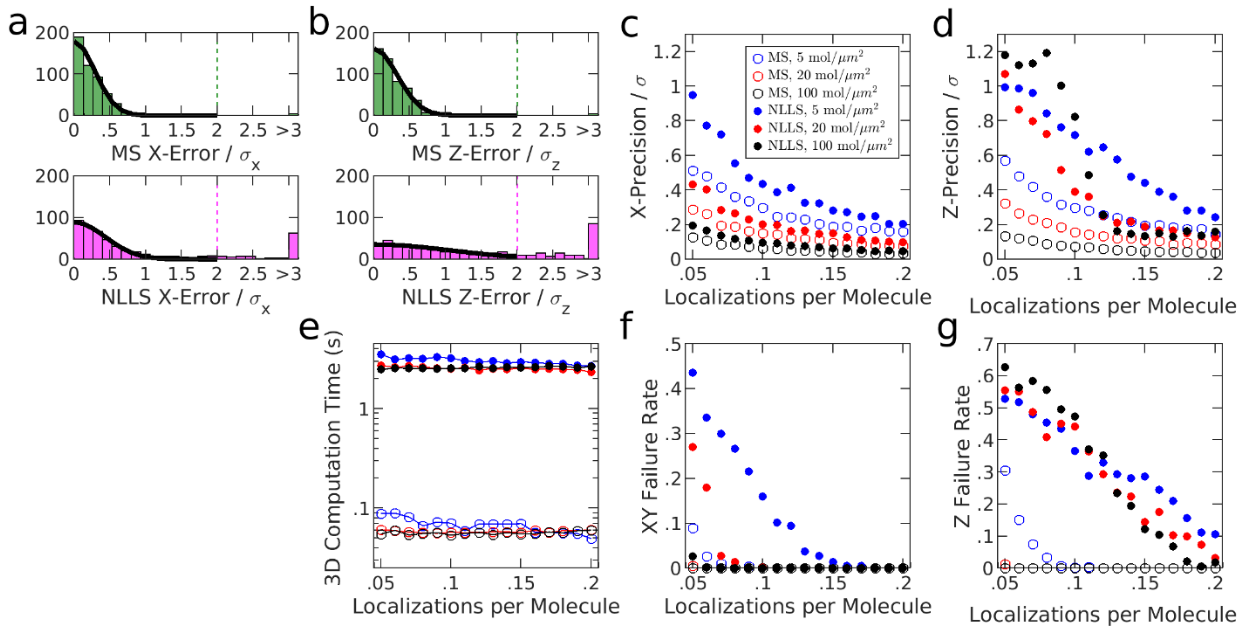

Figure S4. **Evaluating the mean shift (MS) algorithm on 3D simulated data, compared to the NLLS approach.** Simulations and shift determination approaches are described in methods. **a,b)** Histograms of x-errors **(a)** and z-errors **(b)** for the MS and NLLS approaches for the 0.01 molecules/µm^3 and 0.05 localizations per molecule condition. The precision of each method is evaluated for each condition as the standard deviation of a Gaussian fit to the central peak of the histogram. The "failure rate" is the fraction of simulations whose error exceeds twice the localization precision σ, indicated as a dashed line. Three densities are shown in the plots: 5. 20, and 100 molecules/µm^2. **c,d)** Comparison of the lateral **(c)** and axial **(d)** precision of each approach, plotted versus the number of localizations per molecule. **e)** Computation times of the two approaches over the same conditions shown in c,d). **f,g)** Comparison of the failure rate of each approach in lateral **(f)** and axial **(g)** directions under the same range of conditions.

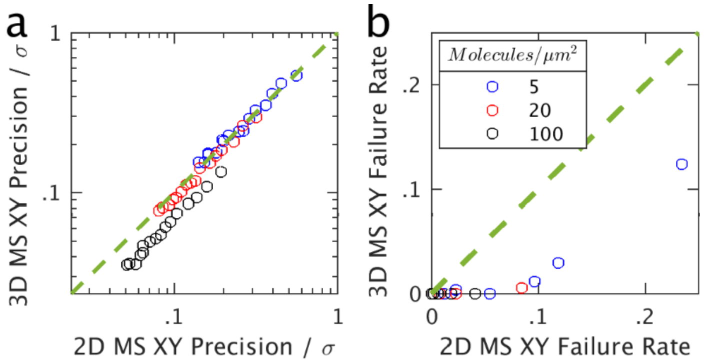

Figure S5. 2D projections of 3D data degrade mean shift (MS) shift estimation performance. Lateral (x-y) precision **(a)** and failure rate **(b)** when MS shift is determined in 3D or in 2D after projecting the localizations into the x-y plane. The points shown each summarize 500 replicates of one simulation condition, with fluorophore density as shown in the legend, and localizations per molecule ranging from .05 to .2. The simulated 3D data used here is constructed such that its 2D projection is identical to the 2D data used for Figure 2.

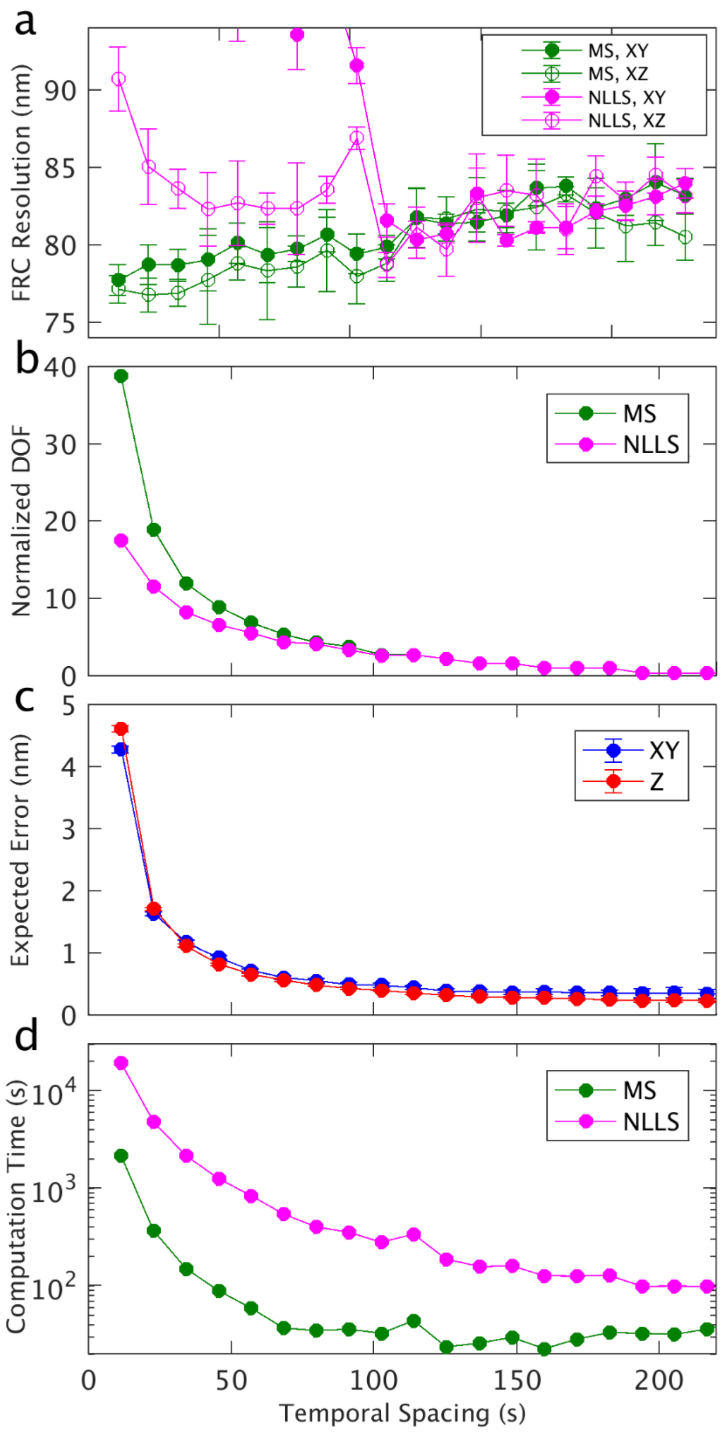

Figure S6. **Drift correction diagnostics for the 3D B cell dataset of Figure 4. a)** FRC resolutions. Error bars are given by the standard deviation over five trials. **b)** The number of degrees of freedom (DOF) after removing outliers (normalized by the number of parameters) for the least square minimization calculation. **c)** RMSE of the lateral (x-y) and axial (z) expected errors for the mean shift calculation of pairwise shifts. Error bars are given by the standard error of the mean. **d)** Total computation time.

Supplemental References

1. Baddeley, A., E. Rubak, and R. Turner. 2016. Spatial point patterns: methodology and applications with R. Boca Raton ; London ; New York: CRC Press, Taylor & Francis Group.

2. Fukunaga, K., and L. Hostetler. 1975. The estimation of the gradient of a density function, with applications in pattern recognition. *IEEE Trans. Inform. Theory*. 21:32–40.

3. Yizong Cheng. 1995. Mean shift, mode seeking, and clustering. *IEEE Transactions on Pattern Analysis and Machine Intelligence*. 17:790–799.

4. Comaniciu, D., and P. Meer. 2002. Mean shift: a robust approach toward feature space analysis. *IEEE Transactions on Pattern Analysis and Machine Intelligence*. 24:603–619.

5. Wang, Y., J. Schnitzbauer, Z. Hu, X. Li, Y. Cheng, Z.-L. Huang, and B. Huang. 2014. Localization events-based sample drift correction for localization microscopy with redundant cross-correlation algorithm. *Opt. Express*. 22:15982.

6. Nieuwenhuizen, R.P.J., K.A. Lidke, M. Bates, D.L. Puig, D. Grünwald, S. Stallinga, and B. Rieger. 2013. Measuring image resolution in optical nanoscopy. *Nat Methods*. 10:557–562.

7. Banterle, N., K.H. Bui, E.A. Lemke, and M. Beck. 2013. Fourier ring correlation as a resolution criterion for super-resolution microscopy. *Journal of Structural Biology*. 183:363–367.

8. Hilgenberg, L.G.W., and M.A. Smith. 2007. Preparation of Dissociated Mouse Cortical Neuron Cultures. *J Vis Exp*.

9. Pappas, S.S., C.-C. Liang, S. Kim, C.O. Rivera, and W.T. Dauer. 2018. TorsinA dysfunction causes persistent neuronal nuclear pore defects. *Human Molecular Genetics*. 27:407–420.

10. Haughton, G., L.W. Arnold, G.A. Bishop, and T.J. Mercolino. 1986. The CH Series of Murine B Cell Lymphomas: Neoplastic Analogues of Ly-1+ Normal B Cells. *Immunological Reviews*. 93:35–52.

11. Stone, M.B., S.A. Shelby, M.F. Núñez, K. Wisser, and S.L. Veatch. 2017. Protein sorting by lipid phase-like domains supports emergent signaling function in B lymphocyte plasma membranes. *eLife Sciences*. 6:e19891.

12. Izeddin, I., J. Boulanger, V. Racine, C.G. Specht, A. Kechkar, D. Nair, A. Triller, D. Choquet, M. Dahan, and J.B. Sibarita. 2012. Wavelet analysis for single molecule localization microscopy. *Opt. Express, OE*. 20:2081–2095.

13. Smith, C.S., N. Joseph, B. Rieger, and K.A. Lidke. 2010. Fast, single-molecule localization that achieves theoretically minimum uncertainty. *Nature Methods*. 7:373–375.

14. Li, Y., M. Mund, P. Hoess, J. Deschamps, U. Matti, B. Nijmeijer, V.J. Sabinina, J. Ellenberg, I. Schoen, and J. Ries. 2018. Real-time 3D single-molecule localization using experimental point spread functions. *Nature Methods*. 15:367–369.
